# Cannabidiol-Based Thiosemicarbazones: A Preliminary Study Evaluating Their Anti-Tyrosinase Properties

**DOI:** 10.3390/molecules30061291

**Published:** 2025-03-13

**Authors:** Eliav Peretz, Noa Ashkenazi, Sanaa Musa

**Affiliations:** 1 Department of Biotechnology, Tel-Hai Academic College, Kiryat Shmona 11016, Israel; 2 Natural Compounds and Organic Synthesis Laboratory, Migal-Galilee Research Institute, Kiryat Shmona 11016, Israel

**Keywords:** cannabidiol, CBD derivatives, thiosemicarbazones, anti-tyrosinase, antioxidant activities

## Abstract

Cannabidiol (CBD), a non-psychoactive cannabinoid, has attracted significant research interest due to its antioxidant, anti-inflammatory, and neuroprotective properties. As a versatile scaffold in drug discovery, CBD has been widely explored for developing novel therapeutics. In this study, we synthesized and evaluated the anti-tyrosinase activity of CBD-based thiosemicarbazones. Structure–activity relationship (SAR) analyses were conducted to assess the impact of various functional groups on tyrosinase inhibition, including an evaluation of inhibitory kinetics for selected compounds. The synthesized derivatives demonstrated potent tyrosinase inhibition, with activity comparable to kojic acid, a standard tyrosinase inhibitor. Given the crucial role of tyrosinase in melanin biosynthesis, these findings suggest that CBD-based thiosemicarbazones could serve as promising candidates for managing tyrosinase-related disorders, including hyperpigmentation and melanogenesis-related conditions. Moreover, the presence of thiosemicarbazone moieties may contribute to the observed inhibitory effects, potentially through metal chelation at the enzyme’s active site. This study provides valuable insights into the design of CBD-derived inhibitors targeting tyrosinase. Further optimization and in-depth biological evaluation are warranted to explore their full therapeutic potential.

## 1. Introduction

Cannabidiol (CBD), a non-psychoactive phytocannabinoid derived from the cannabis plant, has garnered significant attention for its diverse pharmacological properties, including anti-inflammatory, antioxidant, and neuroprotective effects [1,2,3,4,5]. Unlike tetrahydrocannabinol (THC), CBD does not produce psychoactive effects and has been shown to block THC’s influence on the human nervous system [6]. Recent studies suggest that CBD can modulate enzymatic activities, influencing cellular processes such as oxidative stress regulation, inflammation reduction, and tissue regeneration. The discovery of the endocannabinoid system (ECS) in the skin and its pivotal role in maintaining skin homeostasis has further fueled interest in the therapeutic potential of phytocannabinoids for skin-related disorders [7]. Dysregulation of the ECS has been linked to various dermatological conditions, including inflammation, acne, and pigmentation disorders. Consequently, cannabinoids like CBD have emerged as attractive candidates for developing novel CBD derivatives with enhanced properties, offering improved efficacy and specificity in treatments targeting these pathways [8,9,10].

**Scheme 1:**
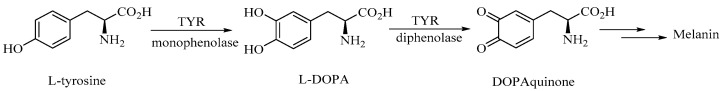
Biosynthetic pathway for melanin production: oxidation of L-tyrosine (monophenol) by tyrosinase (monophenolase) and subsequent oxidation of L-DOPA (o-phenols) to the corresponding quinone (diphenolase).

One critical enzyme involved in skin pigmentation is tyrosinase (EC 1.14.18.1), a copper-containing enzyme belonging to the oxygen oxidoreductase family. Tyrosinase (TYR) serves as the rate-limiting enzyme in melanin biosynthesis, catalyzing two key reactions: the hydroxylation of L-tyrosine to L-3,4-dihydroxyphenylalanine (L-DOPA) (monophenolase activity) and the subsequent oxidation of L-DOPA to dopaquinone (diphenolase activity) (Scheme 1) [11,12]. Melanin plays a crucial protective role by shielding the skin from ultraviolet (UV) radiation, thereby preventing UV-induced damage. Additionally, melanin protects cells by neutralizing reactive oxygen species (ROS). However, the excessive production and accumulation of melanin may lead to hyperpigmentation disorders such as melasma [13], age spots [14], and post-inflammatory hyperpigmentation [15]. Moreover, recent studies have demonstrated that TYR dysregulation has been implicated in the loss of dopaminergic neurons in the brain [16,17]. These conditions not only affect aesthetic appearance but can also impact individuals’ psychological well-being, underscoring the importance of developing effective strategies to regulate TYR activity. As a result, TYR inhibition has been widely studied as a promising approach for controlling melanin production. This strategy holds therapeutic potential for addressing various pigmentation disorders and related conditions.

Traditional TYR inhibitors, such as kojic acid [18], arbutin [19], and hydroquinone [20], have demonstrated efficacy; however, their clinical application is limited due to adverse effects, including skin irritation, genotoxicity, and carcinogenesis potential [1,21,22,23]. This has driven interest in discovering naturally derived and less toxic alternatives. Phytocannabinoids, including CBD, have emerged as potential candidates for interacting with enzymatic pathways in the skin, although their specific impact on TYR activity shows limited activity. Early studies suggest that CBD exhibits moderate TYR inhibitory activity [10,24,25,26]. While structural modifications of CBD have the potential to enhance or diversify its biological properties, they also offer the opportunity to design analogs that enhance selective anti-TYR activity. Investigating the ability of CBD and its derivatives to modulate TYR activity not only expands our understanding of their biochemical properties but also offers potential avenues for developing safer and more effective treatments for pigmentation-related conditions. Given CBD’s promising potential across various diseases, its synthetic modification has garnered significant interest in both academia and the pharmaceutical industry. Researchers aim to enhance its potency, efficacy, and pharmacokinetic properties, making it a valuable target for drug discovery [27,28,29,30]. Synthetic cannabinoids play a crucial role in pharmacological research, particularly in studies on structure–activity relationships (SARs), receptor interactions, and the underlying mechanisms of these compounds [31,32]. However, most research has traditionally focused on altering the pentyl side chain of the olivetolic ring, while modifications at other positions have been relatively unexplored [28,33]. Only recently have studies begun to investigate chemical modifications in the benzene ring of CBD, opening new avenues for structural optimization and therapeutic advancements [27,34,35,36].

Recently, we successfully synthesized and characterized novel aminoguanylhydrazone- and (thio)semicarbazone-based cannabidiol (CBD) compounds. These compounds demonstrated significant antioxidant activity, effectively protecting against LDL oxidation induced by AAPH and copper ions [37]. Notably, it has been suggested that the thiosemicarbazone moiety may act as a copper-chelating agent, potentially inhibiting copper-dependent enzymes such as TYR. Several synthetic thiosemicarbazone derivatives have been reported to exhibit strong TYR-inhibitory properties [38,39,40,41,42,43,44,45]. In this study, we decided to explore the potential of the inhibitory effects of CBD-based thiosemicarbazone analogs on TYR activity, with the aim of designing and synthesizing more potent TYR inhibitors. We evaluate their kinetic parameters and inhibition mechanisms, providing insights into how these compounds interact with the enzyme. By examining the structure–activity relationship (SAR) of CBD derivatives, we aim to elucidate the mechanisms underlying their TYR inhibition and assess their therapeutic potential for managing hyperpigmentation disorders. This exploration could pave the way for the development of safer and more effective treatments targeting pigmentation-related conditions.

## 2. Results

### 2.1. Synthesis

**Figure 1:**
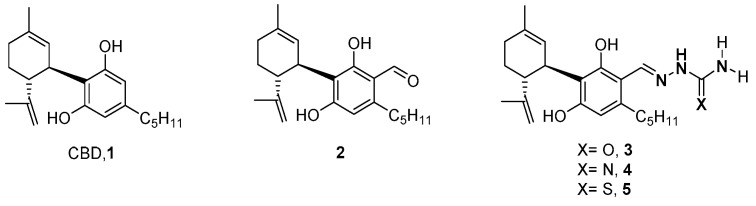
Chemical structures of compounds **1**–**4** [37].

**Scheme 2:**
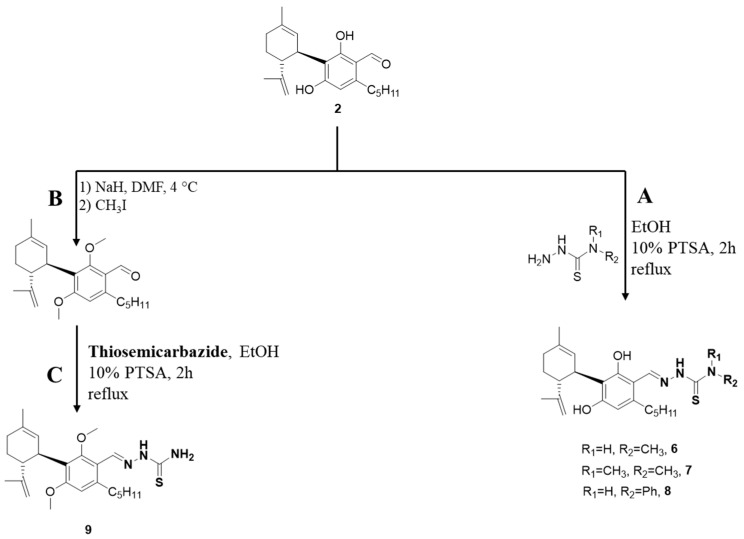
Synthesis pathways of derivatives **5**–**9**. Reagents and conditions: (**A**) *p*-toluene sulfonic acid (PTSA), EtOH, reflux, thiosemicarbazide derivatives (4-methyl-3-thiosemicarbazide (for **6**), 4,4-dimethyl-3-thiosemicarbazide (for **7**), and 4-phenylthiosemicarbazide (for **8**)), 2 h. (**B**) (1) NaH, *N*,*N*-dimethylformamide (DMF), 4 °C. (2) iodomethane, RT, 3 h. (**C**) PTSA, EtOH, reflux, 2 h.

Compounds **1**–**5** were previously synthesized and fully characterized by our group (Figure 1). The thiosemicarbazone–CBD–aldehyde derivatives (**6**–**9**) were successfully synthesized using a standard method under specific reaction conditions, achieving moderate-to-good yields (Scheme 2). All compounds are air-stable solids that have been reliably characterized using standard analytical techniques. The chemical structure of the hydrazone compounds was confirmed through spectroscopic analysis. In the ^1^H-NMR spectra, the imine proton (H–C=NH) exhibits a characteristic singlet resonance at 8.36 and 8.70 ppm across all derivatives. For compounds **5** and **9**, the NH_2_ group appears as two singlets at 7.91 and 8.08 ppm and 7.11 and 8.32 ppm, respectively, indicating magnetically distinct protons due to restricted N–C bond rotation. In compound **6**, the NH group appears as a singlet at 8.27 ppm, which disappears upon substitution to form compound **7**. When the NH_2_ group in **5** is replaced with a N(CH_3_)_2_ group in **7**, the methyl groups appear as two singlets at 3.29 and 3.34 ppm, confirming their magnetic nonequivalence. Similarly, in compound **9**, the two methoxy groups appear as distinct singlets at 3.62 and 3.80 ppm. In contrast, the =N–NH hydrogen consistently appears around 10.89–11.16 ppm in all derivatives, likely due to electron delocalization between the C=N double bond and the thiocarbonyl (C=S) group, which enhances resonance effects and further deshields the NH proton.

The FTIR spectra of compounds **5**–**9** (Figure S3) confirmed the presence of symmetric and asymmetric NH_2_ stretching vibrations in compounds **5**, **6**, **8**, and **9**, observed in the 3500–3140 cm^−1^ region. In contrast, compound **7**, which lacks this functional group and contains a disubstituted amine, did not exhibit a prominent peak in this range. The characteristic C=N stretching vibration of the imine group was detected near 1590 cm^−1^ as a strong peak, while a strong C–N stretching vibration near 1270 cm^−1^ further supported the structural integrity of the synthesized compounds. Additionally, all compounds exhibited a C=S stretching vibration around 1400 cm^−1^ and a C=S bending vibration near 888 cm^−1^, along with a N–N stretching vibration of medium–strong intensity around 1100 cm^−1^. Together, these spectral data confirm the successful formation of the expected products [46,47].

### 2.2. Antioxidant Activity by FRAP and DPPH Assays

**Figure 2:**
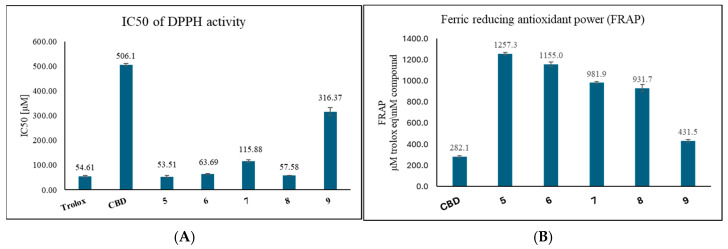
(**A**) IC_50_ of the tested compounds (CBD and **5**–**9**) in DPPH radical scavenging activity. IC_50_ values are expressed as mean ± SD of the three independent experiments in triplicates. (**B**) Ferric-reducing antioxidant power (FRAP) assays for estimating antioxidant activities of the tested compounds (**5**, **6**, **7**, **8**, **9**, and CBD). Data are presented in trolox equivalent (TE) (1 mM of the tested compound is equivalent to X µM (values shown as bars) of trolox).

The antioxidant properties of the thiosemicarbazone–CBD–aldehyde derivatives (**5**–**9**) were evaluated using two methods: FRAP and DPPH assays. The analysis revealed a strong reducing activity of the newly synthesized compounds (**5**–**9**) in both tests. The antioxidant activity was compared to that of trolox. In the 1,1-diphenyl-2-picryl-hydrazyl (DPPH), IC_50_ values were 54.61 ± 3.45 μM for trolox and 53.51 ± 5.29, 63.69 ± 3.29, 115.88 ± 5.05, 57.58 ± 1.71, and 316.37 ± 15.95 μM for **5**, **6**, **7**, **8**, and **9**, respectively (Figure 2A). IC_50_ corresponds to the concentration of tested compounds that is able to scavenge 50% of the initial DPPH radicals. Low IC_50_ values indicate high antioxidant activity [48]. Compounds **5** and **8** show the highest DPPH radical-scavenging activity, which is similar to the activity of the trolox. Compounds **6** and **7** show lower scavenging activity than compound **5**. Notably, all thiosemicarbazone–CBD–aldehyde derivatives exhibited significantly higher antioxidant activity than CBD (IC_50_ = 506.10 ± 4.67 μM).

The ferric-reducing antioxidant potential (FRAP) assay is a simple and inexpensive protocol that is particularly helpful in assessing the antioxidant power of a sample in which those constituents that are present act by reducing ions or by donating an electron and not by the radical quenching mechanism [49]. A higher FRAP value indicates greater antioxidant activity. Among the tested compounds, compound **5** exhibited the highest antioxidant activity, as determined by the FRAP method (1257 ± 6.78 μM TE). In comparison, compounds **6**–**9** displayed lower activity, with values of 1115.0 ± 10.12, 981.9 ± 9.66, 931.7 ± 8.97, and 431.5 ± 13.51 μM TE, respectively. Notably, these values remain approximately twice as high as the FRAP activity of CBD (282.1 ± 5.68 μM TE) (Figure 2B).

### 2.3. Inhibition of TYR Activity

The inhibitory effects of the thiosemicarbazone–CBD–aldehyde derivatives on the diphenolase activity of mushroom TYR were assessed by UV-Vis spectrometry using L-DOPA as the substrate. The anti-TYR activity was evaluated by determining the IC_50_ values, which are defined as the concentration of the inhibitor able to halve the maximum reaction rate in the adopted experimental conditions, and were calculated from the logarithmic concentration–inhibition curves of the thiosemicarbazone–CBD–aldehyde derivatives.

The results, summarized in Table 1, indicate that the synthesized compounds inhibited the diphenolase activity of TYR in a dose-dependent manner. Kojic acid (KA), a well-known and extensively studied tyrosinase inhibitor, served as a positive control in these experiments [50,51]. Notably, variations in IC_50_ values for KA have been reported, which can be attributed to differences in TYR sources and experimental conditions [52]. Compounds **1**–**4**, including CBD, exhibited minimal inhibitory activity against mushroom TYR, with IC_50_ values > 100 μM. In contrast, the thiosemicarbazone–CBD derivatives (**5**–**9**) demonstrated significant inhibition, with IC_50_ values of 27.72, 29.38, 28.07, 42.16, and 22.41 μM, respectively, compared to 35.33 μM for KA. These findings suggest that the thiosemicarbazone moiety enhances the TYR inhibitory activity of CBD derivatives.

**Figure 3:**
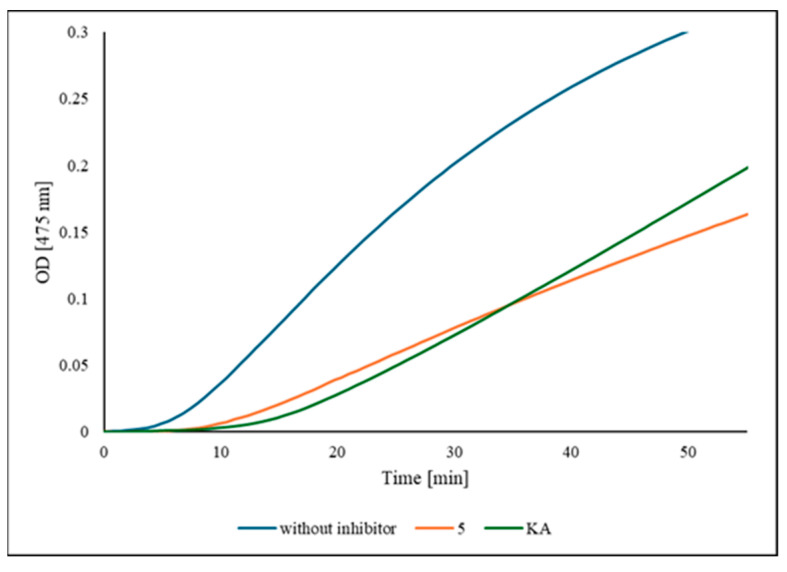
The time-dependent changes in OD at 475 nm correspond to the formation of L-dopaquinone. Blue line: control. Orange line: 20 μM of compound **5**. Green line: 20 μM of KA.

Additionally, the impact of the newly synthesized compounds on the initial stage of TYR enzymatic activity was evaluated using L-tyrosine as the substrate. This initial phase is characterized by a lag period and an increased reaction rate. Figure 3 illustrates the change in optical density (OD) at 475 nm over time, corresponding to o-quinone formation, in the control, and the presence of compound **5** and KA. In the control, the lag phase was 264 s (4.40 min), which extended to 593 sec (9.89 min) with the addition of compound **5** and to 678 s (11.31 min) with KA. The lag times for compounds **6**–**9** are summarized in Table 2, showing extensions to 363 (6.06), 371 (6.19), 274 (4.58), and 400 (6.67) s (min), respectively. Notably, compounds **1**–**4**, including CBD, did not exhibit any effect on extending the lag phase of TYR activity.

**Figure 4:**
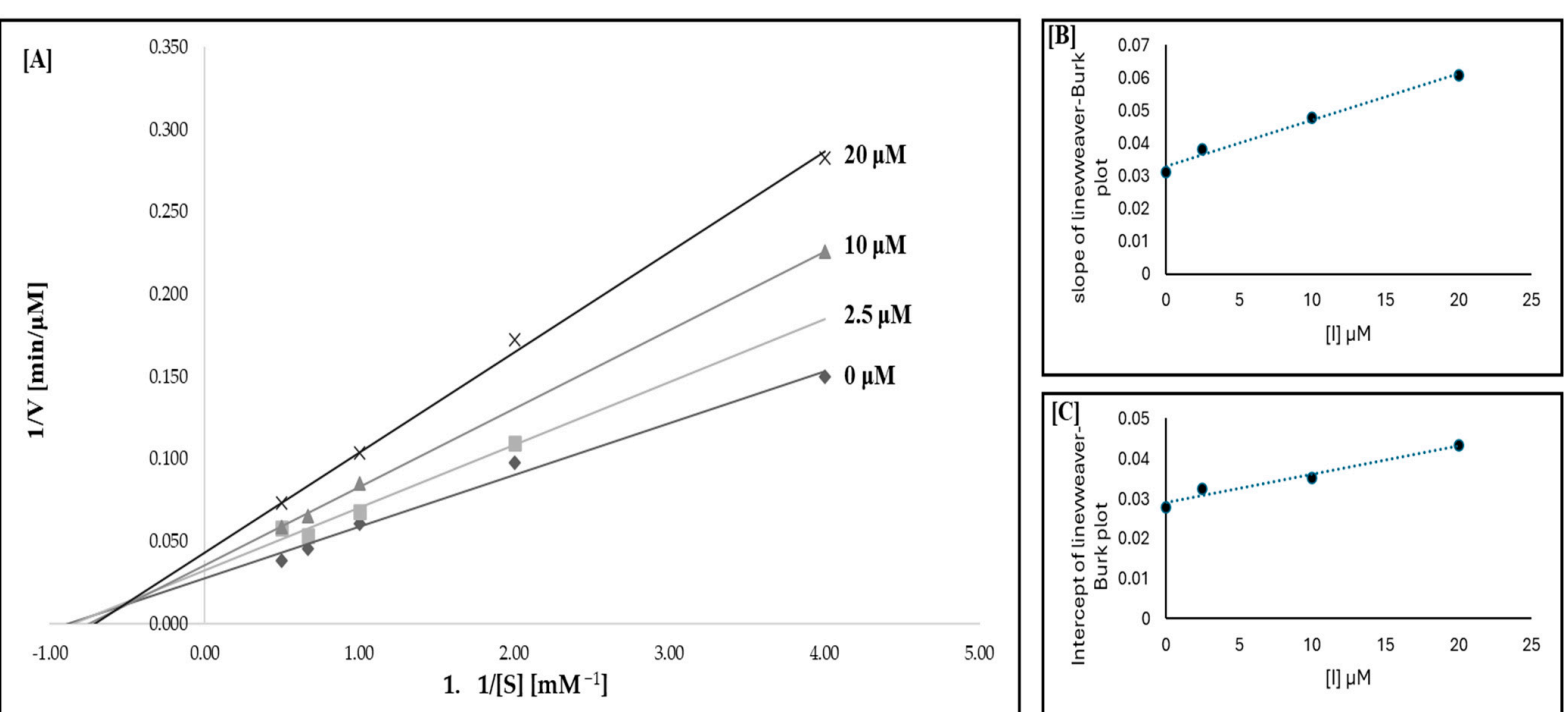
Tyrosinase activity for compound **5**: (**A**) Lineweaver–Burk plot; (**B**) slope of the Lineweaver–Burk plot as a function of inhibitor concentration; (**C**) intercept of the Lineweaver–Burk plot as a function of inhibitor concentration.

The inhibition mechanism of compound **5** on the diphenolase activity of TYR was further studied. The inhibition type was determined by the classical Lineweaver–Burk plot based on the inhibitory results of **5**. As shown in Figure 4, the Lineweaver–Burk plot of 1/v versus 1/[S] in the presence of different concentrations of each inhibitor gave a family of straight lines with different slopes. The resulting plot showed straight lines intersecting in the second quadrant, indicating that, as the concentration of compound **5** increased, the maximum reaction velocity (Vₘₐₓ) decreased while the Michaelis constant (Kₘ) increased. Such behavior is characteristic of mixed-type inhibition, suggesting that **5** can bind to both the free and enzyme–substrate complex.

### 2.4. Spectrofluorometric Analysis for the Interactions Between Compound ***5*** and TYR

**Figure 5:**
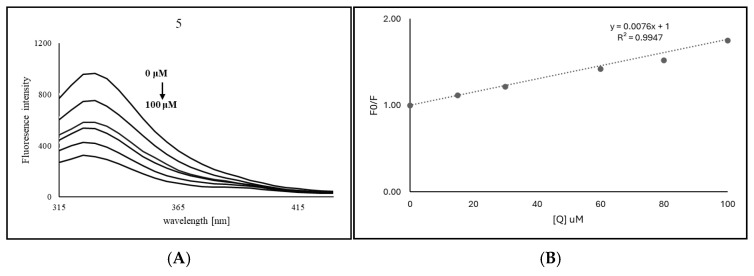
(**A**) Fluorescence quenching of TYR at λ_ex_ = 280 nm in increasing concentrations of **5** at 310 K. (**B**) Stern–Volmer plot.

Figure 5 illustrates the effects of various concentrations of compound **5** on the intrinsic fluorescence of mushroom TYR. This fluorescence quenching method has been widely used to study interactions between fluorescent biomacromolecules and small molecules. In an aqueous solution, TYR exhibits strong fluorescence, with a maximum emission at 325 nm when excited at 280 nm, primarily due to its tryptophan (Trp) residues. The fluorescence spectrum of compound **5** does not overlap with that of TYR. Upon the gradual addition of compound **5**, a significant decrease in TYR’s fluorescence intensity was observed, while the emission peak position remained unchanged. This suggests that compound **5** interacts with TYR, leading to fluorescence quenching without altering the enzyme’s conformation [53]. Similar fluorescence quenching profiles and linear Stern–Volmer plots were observed for compounds **6**–**9** (Figure S5).

### 2.5. Copper Ion Chelation

**Figure 6:**
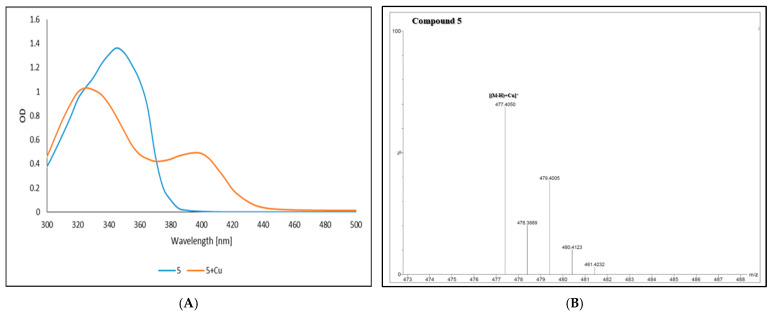
(**A**) UV-Vis spectrum of compound **5** (blue line) and copper complex with **5** (orange line). (**B**) ESI-MS of a copper complex with **5**.

The UV-Vis analysis of free thiosemicarbazone derivatives and their interactions with copper ions was performed in ethanol–PBS within the 280–500 nm range at room temperature. The free ligands exhibited characteristic absorption bands at 300 nm and 342 nm, corresponding to π-π* transitions in the aromatic moiety and n-π* transitions in the thiosemicarbazone group, respectively (Figure 6A, blue line). Upon interaction with copper ions, a new band around 400 nm was obtained, attributed to ligand-to-metal charge transfer (LMCT) (Figure 6A, orange line). This band is associated with S→Cu, N→Cu, and O→Cu charge transfer, providing further evidence for the coordination of the ligands to the metal center, forming a tridentate coordination mode [54]. In compound **9**, the UV-Vis spectrum exhibited a weaker absorption band around 400 nm, indicating a distinct coordination profile compared to other compounds. This weaker band suggests that the interaction between the methoxy group (OCH_3_) and the copper ion is less cooperative, resulting in reduced LMCT and different coordination behavior.

The copper complex formation with compound **5** was characterized using the ESI-MS technique. The spectrum displayed a prominent peak at 477 *m*/*z*, indicating the formation of the copper complex with compound **5** (Figure 6B). Likewise, compounds **6**–**9** exhibited mass peaks at 491, 505, 553, and 506 *m*/*z*, respectively, corresponding to the formation of copper complexes (Figure S7). These masses indicate the presence of [(M − H) + Cu]^+^ species.

**Figure 7:**
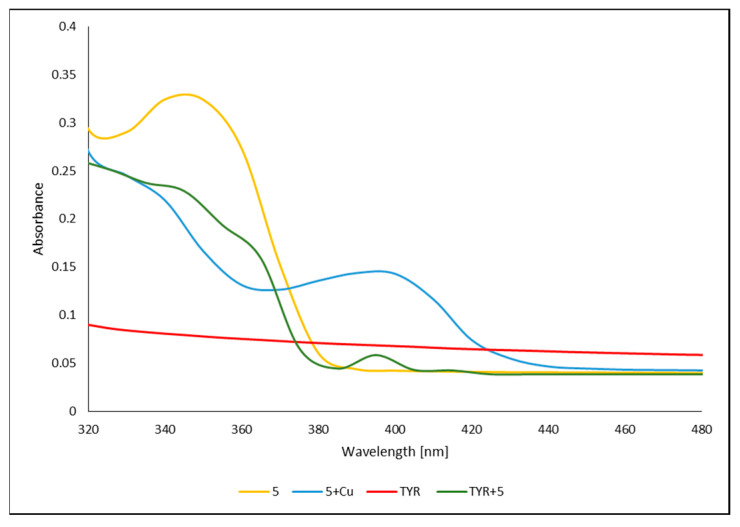
UV-Vis spectra of compound **5** and TYR under different conditions: Compound **5** with and without copper ions (blue and yellow lines, respectively), and TYR with and without compound **5** (green and red lines, respectively).

Furthermore, the UV-Vis spectrum was recorded after incubating compound **5** with TYR for 30 min at 37 °C (Figure 7). As expected, the UV-Vis spectrum of TYR alone showed no absorption in the 320–500 nm range (Figure 7, red line). However, upon incubation with compound **5**, the spectrum shifted (Figure 7, green line), closely resembling the copper complex spectrum (Figure 7, blue line) rather than the free 5 spectrum (Figure 7, yellow line). A minor absorption of around 400 nm was observed, which likely indicates an LMCT interaction between the copper ion of TYR and the thiosemicarbazone moiety of **5**.

## 3. Discussion

The development of TYR inhibitors remains a focal point in both scientific research and industrial applications due to their potential in treating hyperpigmentation disorders and their utility in cosmetic formulations [55,56]. Concurrently, there is a growing interest in the synthesis of novel cannabinoid derivatives to evaluate their diverse biological activities. CBD, in particular, has garnered significant attention for its therapeutic potential, prompting medicinal chemists to explore its chemical framework for drug development. Recent studies have investigated the anti-melanogenic and anti-TYR properties of CBD and minor phytocannabinoids, expanding the potential applications of these compounds in skin-related treatments [9,25,26].

As a part of our research, in this work, we synthesized and characterized new thiosemicarbazone–CBD–aldehyde derivatives (compounds **6**–**9**), building upon our previously synthesized compounds **1**–**5**. These novel derivatives were obtained through standard methods under specific reaction conditions, yielding moderate-to-good yields. The chemical structures of the synthesized compounds were confirmed using various spectroscopic techniques, including NMR, FTIR, and mass spectrometry. Our findings contribute to expanding the research on CBD derivatives and their potential biological activities.

The antioxidant properties of the newly synthesized thiosemicarbazone–CBD–aldehyde derivatives were evaluated through antiradical and ferric-reducing assays, with trolox serving as the standard. The results demonstrated that these compounds exhibit significant antioxidant activity. The scavenging effect is attributed to the N–H group in the thiosemicarbazone moiety and the O–H groups of the CBD skeleton, which can donate hydrogen atoms to free radicals. Upon donating a hydrogen atom, the compounds form a radical that delocalizes onto the benzene ring of the CBD, resulting in a stable resonance hybrid [57]. Among the derivatives, compound **5** exhibited the highest antioxidant potential. The IC_50_ values for compounds **6** and **7** were 63.69 and 115.88 μM, respectively, compared to **5** with an IC_50_ of 53.51 μM. These findings suggest that substituting the NH_2_ group with electron-donating groups (such as methyl in **6** or dimethyl in **7**) reduces the scavenging ability of the DPPH free radical. In contrast, substitution with a phenyl group resulted in a similar activity to **5** (IC_50_ of 57.58 μM for compound **8**), likely due to the phenyl group enhancing radical stability compared to methyl or dimethyl groups [58]. Compound **9** displayed lower scavenging activity than **5**–**8**, probably due to substituting the OH groups with dimethyl ether groups. Collectively, these studies suggest that incorporating thiosemicarbazone moieties into the CBD framework enhances its antioxidant properties, making these derivatives promising candidates for therapeutic applications targeting oxidative-stress-related conditions. On the other hand, the FRAP assay showed similar activity for compounds **5**–**9**, indicating significantly better ferric-reducing activity than CBD. This suggests that the presence of the thiosemicarbazone moiety in the CBD framework plays a crucial role in enhancing antioxidant activity.

To evaluate the TYR-inhibitory properties of our compounds, we utilized mushroom TYR (*Agaricus bisporus*) as a model system, as it shares a 23% amino acid identity with human TYR and is widely used for studying mammalian TYR due to its high homology [59]. The anti-TYR activity of compounds **1**–**9** was tested, with compounds **1**–**4** showing a low inhibitory effect on TYR activity in mushroom TYR’s monophenolase and diphenolase activities. As can be observed in Table 1, the semicarbazone and aminoguanidyl hydrazone derivatives (compounds **3** and **4**, respectively) did not exhibit any inhibitory activity, suggesting that the thiosemicarbazone moiety is crucial for the activity. Inhibition likely occurs through the coordination of copper ions present in the active site of TYR by the C=S group. Some studies have indicated that TYR inhibitor activity is reduced when sulfur is substituted with an oxygen atom [60,61]. The sulfur atom in thiosemicarbazones interacts more effectively with the copper ion in the TYR active site than the oxygen and nitrogen atoms in analogous derivatives.

In contrast, compounds **5**–**9** were found to be more effective or similar to that for KA. SAR analysis for compounds **5**–**9** in diphenolase activity revealed that substituting the hydrogen of the NH_2_ group with methyl, dimethyl, or phenyl groups reduces TYR inhibition. While the methyl and dimethyl substitutions (compounds **6** and **7**, respectively) only slightly decrease the inhibitory effect, the phenyl group reduces the inhibitory effect by about two-fold, most likely due to steric hindrance. However, compound **9** exhibited a better inhibitory effect than **5** and KA. All these considerations suggest that, among the structural modifications made in the studied compounds, introducing sulfur and NH_2_ groups in the thiosemicarbazone moiety could be a promising strategy to improve the efficacy of tyrosinase inhibitors. When the monophenolase activity of TYR, using L-tyrosine as the substrate, was tested with thiosemicarbazone derivatives (**5**–**9**), an extension of the lag phase was observed. Compound **5** notably increased the lag phase approximately two-fold. This observation suggests that the thiosemicarbazone derivatives may interact with the enzyme in a way that influences the enzyme’s activity, potentially through a reducing effect on the copper ions at the active site. The extension of the lag phase indicates a delay in the enzyme’s catalytic action, possibly due to the binding or modification of the enzyme’s active site by the derivatives. More detailed studies would be needed to elucidate the precise mechanism behind this effect.

Kinetic analyses of the diphenolase activity of TYR demonstrated that compound **5** is a reversible, mixed-type inhibitor of mushroom TYR (Figure 4). The Lineweaver–Burk plot revealed straight lines with varying slopes that intersected in the second quadrant. As the concentration of compound **5** increased, the v_max_ value decreased while the k_m_ value increased. This behavior confirms that **5** is a mixed-type inhibitor, capable of binding not only to the free enzyme but also to the enzyme–substrate complex, thereby affecting the TYR-L-DOPA affinity upon inhibitor binding. When compounds **5**–**9** were incubated with TYR for 3 h and subsequently dialyzed, the enzyme’s activity was restored to levels similar to the control enzyme that had not been exposed to the inhibitors. Additionally, ESI-MS analysis of the inhibitors revealed no changes in their mass during the incubation period, and no oxidation was observed. These results suggest that the inhibitors suppress TYR activity by chelating the copper ions at the enzyme’s active site without undergoing structural changes themselves.

Moreover, fluorescence quenching experiments were conducted to analyze the interactions between compounds **5**–**9** and TYR (Figure 5 and Figure S5). As shown in Figure 5, the fluorescence emission spectra of TYR at 310 K decreased significantly in a dose-dependent manner with the gradual addition of **5**. However, the maximum emission peak remained unchanged, indicating that **5** interacts with Trp residues or nearby amino acids in mushroom TYR without significantly affecting the polarity of the microenvironment around Trp. The Stern–Volmer plots (Figure 5B) derived from the fluorescence quenching data exhibited good linearity, indicating a single quenching mechanism. The quenching constants were determined to elucidate the quenching process (Table 3). Fluorescence quenching in proteins can be categorized as either dynamic or static depending on the type of interaction between the quencher and the protein [62]. It was reported that the limiting fluorescence quenching rate constant of biological macromolecules with various quenchers in an aqueous medium via diffusion collision is about 2 × 10^10^ M^−1^·s^−1^. If k_q_ is greater than this value, the quenching process is predominantly static, involving the formation of a stable complex. As shown in Table 3, the k_q_ values for compounds **5**–**9** binding to TYR exceeded 2 × 10^10^ M^−1^·s^−1^, indicating that static quenching is the dominant mechanism.

The K_a_ values for the binding of thiosemicarbazone–CBD derivatives with TYR ranged from 0.52 × 10^4^ to 2.55 × 10^4^ M^−1^. Notably, the K_a_ values of compounds **5** and **9** were similar, both exhibiting high inhibitory potency against TYR (IC_50_ = 27.72 and 22.41 μM for **5** and **9**, respectively), suggesting that the NH_2_ group plays a crucial role in TYR inhibition. In contrast, compound **8** displayed the highest K_a_ value but the lowest inhibitory potency (IC_50_ = 42.16 μM). These observations suggest that the presence of a phenyl group may enhance hydrophobic or π-π stacking interactions with the TYR environment, stabilizing the TYR–quencher complex. However, high K_a_ values do not always correlate with effective inhibition, potentially because binding alone may not sufficiently disrupt the enzyme’s catalytic function [63,64].

**Figure 8:**
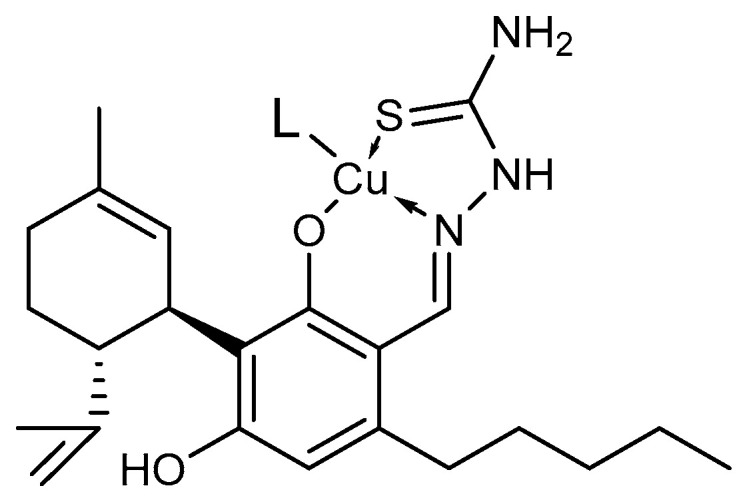
Proposed chemical structure of the complex formed between compound **5** and the copper ion.

The interactions between the inhibitors and copper ions were evaluated using UV-Vis spectroscopy at pH 6.8. The formation of complexes between the tested compounds and copper ions led to a bathochromic (red) shift in the UV-Vis spectrum, with a new peak appearing around 400 nm (Figure 5A and Figure S6). This shift indicates the donation of lone pairs from the nitrogen atoms of the azomethine group in the Schiff base to the copper ions, suggesting an LMCT. Notably, compound **9** displayed a distinct UV-Vis profile compared to compounds **5**–**8**, highlighting the critical role of the hydroxyl group (OH) of the phenol in contributing to the coordination with copper. It is suggested that the copper complex adopts a tridentate SNO coordination mode with the thiosemicarbazone derivatives, leading to the formation of thermodynamically stable five- and six-membered conjugate rings (Figure 8). Upon the addition of 100 μM ethylenediaminetetraacetic acid (EDTA), the spectrum reverted to that of the free ligand, confirming the displacement of the compounds from the copper ions by EDTA. When compound **5** was incubated with TYR, a change in its UV-Vis spectrum was observed, suggesting an interaction between the copper ion in TYR’s active site and the thiosemicarbazone moiety of **5** (Figure 7). To gain a deeper understanding of this interaction, further confirmation through ongoing docking studies is being conducted, with results to be published in the near future. Moreover, the ESI-MS analysis of the copper complex formed with **5** revealed a peak at *m*/*z* 477, corresponding to the chelation of copper (Figure 6B). The observed isotopic pattern further confirmed the formation of the copper complex, as it matched the characteristic distribution expected for copper-containing species. This isotopic signature is a reliable indicator of metal–ligand complexation in mass spectrometric studies. All the thiosemicarbazone–CBD–aldehyde derivatives (**6**–**9**) also exhibited new peaks, with masses and patterns consistent with copper chelation, as well as characteristic absorption changes in UV-Vis spectra (Figure S7).

## 4. Materials and Methods

### 4.1. General

All chemicals and reagents were purchased from Sigma-Aldrich (Rehovot, Israel), and Rhenium, Modi’in-Maccabim-Re’ut, Israel. Flash column chromatography was performed with Merck ultra-pure silica gel (Darmstadt, Germany, 230–400 mesh). Yields refer to isolated compounds greater than 95% purity as determined by proton nuclear magnetic resonance spectroscopy (NMR, Brucker, 400 MHz, Bruker Corporation, Billerica, MA, USA), high-performance liquid chromatography coupled with a photodiode array detector (HPLC, Agilent 1290, Agilent Technologies, Inc., Santa Clara, CA, USA), and gas chromatography–mass spectrometry (GC-Agilent 7890A, MS-Agilent 5975C, Agilent Technologies, Inc., Santa Clara, CA, USA) analysis. FTIR spectra were recorded by the Nicolet™ iS™ 10 FTIR Spectrometer (Thermo Scientific™, Waltham, MA, USA). UV-VIS spectra were recorded by the Tecan Infinite 200 PRO spectrophotometer (Mennedorf, Switzerland). The preparation of compounds **1**–**5** was carried out in accordance with the procedures described in our previous publication [37]. For complete analytical data, please refer to the Supplementary Materials.

### 4.2. Preparation of 3-Formyl Cannabidiol Dimethyl Ether

NaH (1.16 mmol, 46 mg) was gradually added to a cooled solution of compound **2** (0.58 mmol, 200 mg) in *N*,*N*-dimethylformamide (DMF). The mixture was stirred in an ice bath for 30 min before iodomethane (1.16 mmol, 75 μL) was added dropwise. Stirring continued at this temperature for 15 min, followed by warming to room temperature and further stirring for 3 h. The reaction progress was monitored using GC-MS. Upon completion, the product was extracted with ethyl acetate, dried over Na_2_SO_4_, evaporated to dryness, and used in the next step without further purification. GCMS, *m*/*z*: 370 (M), 355 (M-CH_3_), 339 (M-OCH_3_) (Figure S1).

### 4.3. General Procedure for the Synthesis of Thiosemicarbazone–Cannabidiol–Aldehyde Derivatives

Thiosemicarbazide derivative (0.31 mmol), aldehyde derivative (100 mg, 0.29 mmol), and PTSA (10% mole) were dissolved in 5 mL of absolute ethanol. The reaction mixture was refluxed for 2 h under an inert atmosphere. The reaction completion and formation of the product were confirmed using thin-layer chromatography. The solvent was evaporated to dryness. The residue was extracted with dichloromethane and saturated bicarbonate solution, dried over Na_2_SO_4_, filtered, and evaporated to dryness.

The 3-thiosemicarbazone–CBD–aldehyde (**5**) was purified by chromatography using a silica gel column eluted with 15% ethyl acetate in hexane to give a white solid (79 mg, 65% yield). The product was identified by LC-MS and NMR analyses. ^1^H-NMR (DMSO-*d*_6_, 400 MHz), δ: 11.21 (1H, s), 10.39 (1H, bs), 9.67 (1H, bs), 8.57 (1H, s), 8.08 (1H, bs), 7.91 (1H, bs), 6.24 (1H, s), 5.16 (1H, s), 4.56 (1H, s), 4.47 (1H, s), 3.98 (1H, d), 3.17 (1H, t), 2.57 (2H, m), 2.19 (1H, m), 2.02 (1H, m), 1.71 (8H, m), 1.50 (2H, m), 1.36 (4H, m), 0.93 (3H, t). ^13^C-NMR (DMSO-*d*_6_), δ: 177.89, 170.79, 149.30, 146.82, 142.63, 131.13, 126.52, 115.44, 110.44, 107.42, 43.75, 35.92, 32.93, 31.77, 31.71, 30.74, 29.84, 23.76, 22.46, 21.22, 19.56, 14.45. FTIR (cm^−1^): 3429, 3140 (N–H stretching), 2923 (aromatic C–H stretching), 1586 (C=N), 1525 (C=C), 1275 (C–N), 1431 (C=S), 1152 (N–N). $\alpha_{598\;\mathrm{nm}}^{25\;{}^{\circ}C}(\mathrm{EtOH})=-90.54$. mp = 120–124 °C. LC-MS (ESI^+^) *m*/*z* = 416.2371 (M + 1). λ_max_ (nm), ε (L·cm^−1^·mol^−1^) = 345 (6807). HPLC purity > 97%.

The 3-(4-methyl-3-thiosemicarbazone)–CBD–aldehyde (**6**) was purified by chromatography using a silica gel column eluted with 15% ethyl acetate in hexane to give a white solid (76 mg, 61% yield). The product was identified by LC-MS and NMR analyses. ^1^H-NMR (DMSO-*d*_6_, 400 MHz), δ: 11.16 (1H, s), 9.61 (1H, bs), 8.52 (1H, s), 8.27 (1H, bs), 6.19 (1H, bs), 5.12 (1H, s), 4.51 (1H, s), 4.42 (1H, s), 3.96 (1H, d), 3.34 (3H, s), 3.13 (1H, m), 2.14 (1H, m), 1.98 (1H, m), 1.68 (8H, m), 1.46 (2H, m), 1.31 (4H, m), 0.88 (3H, t). ^13^C-NMR (DMSO-*d*_6_), δ: 176.96, 149.33, 146.55, 142.52, 131.13, 126.62, 115.39, 110.35, 43.68, 35.99, 32.95, 31.71, 30.74, 29.89, 23.78, 22.47, 19.65, 14.45. FTIR (cm^−1^): 3429, 3211 (N–H stretching), 2926 (aromatic C–H stretching), 1584 (C=N), 1537 (C=C), 1260 (C–N), 1440 (C=S), 1103 (N–N). $\alpha_{598\;\mathrm{nm}}^{25\;{}^{\circ}C}\left(\mathrm{EtOH}\right)=-103.73$. mp = 127–132 °C. LC-MS (ESI^+^) *m*/*z* = 430.80 (M + 1). λ_max_ (nm), ε (L·cm^−1^·mol^−1^) = 345 (7729). HPLC purity > 97%.

The 3-(4,4-dimethyl-3-thiosemicarbazone)–CBD–aldehyde (**7**) was purified by chromatography using a silica gel column eluted with 15% ethyl acetate in hexane to give a white solid (87 mg, 71% yield). The product was identified by LC-MS and NMR analyses. ^1^H-NMR (DMSO-*d*_6_, 400 MHz), δ: 10.89 (1H, bs), 9.41 (1H, bs), 8.70 (1H, s), 6.16 (1H, s), 5.11 (1H, s), 4.49 (1H, s), 4.41 (1H, s), 3.96 (1H, m), 3.34 (3H, s), 3.29 (3H, s), 3.11 (1H, s), 2.14 (1H, m), 1.97 (1H, m), 1.73 (10H, m), 1.31 (4H, m), 0.88 (3H, t). ^13^C-NMR (DMSO-*d*_6_), δ: 176.55, 149.59, 145.61, 140.56, 130.66, 127.19, 115.42, 110.13, 108.28, 43.76, 35.91, 32.63, 31.66, 31.33, 30.80, 30.00, 23.79, 22.52, 19.76, 14.43. FTIR (cm^−1^): 3146 (N–H stretching), 2926 (aromatic C–H stretching), 1590 (C=N), 1539 (C=C), 1256 (C–N), 1436 (C=S), 1117 (C–N). $\alpha_{598\;\mathrm{nm}}^{25\;{}^{\circ}C}(\mathrm{EtOH})=-113.34$. mp = 115–120 °C. LC-MS (ESI^+^) *m*/*z* = 444.2680 (M + 1). λ_max_ (nm), ε (L·cm^−1^·mol^−1^) = 340 (5236). HPLC purity > 97%.

The 3-(4-phenyl-3-thiosemicarbazone)–CBD–aldehyde (**8**) was purified by chromatography using a silica gel column eluted with 15% ethyl acetate in hexane to give a yellow solid (109 mg, 76% yield). The product was identified by LC-MS and NMR analyses. ^1^H-NMR (DMSO-*d*_6_, 400 MHz), δ: 11.50 (1H, s), 10.06 (1H, s), 9.66 (1H, bs), 8.61 (1H, s), 7.46 (2H, d, J = 8Hz), 7.36 (2H, t, J = 8Hz), 7.19 (1H, t, J = 8 Hz), 6.21 (1H, s), 5.12 (1H, s), 4.50 (1H, s), 4.43 (1H, s), 3.96 (1H, d, J = 12 Hz), 3.14 (1H, t, J = 12 Hz), 2.12 (1H, m), 1.96 (1H, m), 1.64 (8H, m), 1.50 (2H, m), 1.32 (4H, m), 0.98 (3H, t). ^13^C-NMR (DMSO-*d*_6_), δ: 175.96, 149.30, 147.23, 146.75, 140.00, 142.52, 131.18, 128.57, 126.55, 126.14, 125.53, 115.54, 110.35, 43.74, 35.99, 33.00, 31.71, 30.74, 29.86, 23.78, 22.51, 19.64, 14.47. FTIR (cm^−1^): 3327, 3153 (N–H stretching), 2920 (aromatic C–H stretching), 1582 (C=N), 1526 (C=C), 1259 (C–N), 1443 (C=S), 1105 (N–N). $\alpha_{598\;\mathrm{nm}}^{25\;{}^{\circ}C}\left(\mathrm{EtOH}\right)=-98.65$. mp = 166–173 °C. LC-MS (ESI^+^) *m*/*z* = 492.2681 (M + 1). λ_max_ (nm), ε (L·cm^−1^·mol^−1^) = 355 (6827). HPLC purity > 96%.

The 3-thiosemicarbazone–CBD–dimethylether (**9**) was purified by chromatography using a silica gel column eluted with 15% ethyl acetate in hexane to give a white solid (67 mg, 52% yield). The product was identified by LC-MS and NMR analyses. ^1^H-NMR (DMSO-*d*_6_, 400 MHz), δ: 11.46 (1H, s), 8.36 (1H, s), 8.32 (1H, s), 7.11 (1H, s), 6.69 (1H, s), 5.21 (1H, s), 4.47 (1H, s), 4.45 (1H, s), 3.85 (2H, m), 3.80 (3H, s), 3.62 (3H, s), 2.94 (2H, m), 2.20 (1H, m), 2.02 (1H, m), 1.71 (8H, m), 1.50 (2H, m), 1.34 (4H, m), 0.92 (3H, t). ^13^C-NMR (DMSO-*d*_6_), δ: 177.96, 148.80, 142.61, 142.25, 131.92, 123.78, 110.76, 63.27, 56.23, 34.477, 32.02, 30.71, 30.64, 29.44, 23.73, 22.55, 22.50, 19.53, 14.43. FTIR (cm^−1^): 3429, 3140 (N–H stretching), 2920 (aromatic C–H stretching), 1587 (C=N), 1528 (C=C), 1257 (C–N), 1450 (C=S), 1106 (N–N). $\alpha_{598\;\mathrm{nm}}^{25{}^{\circ}C}(\mathrm{EtOH})=-105.35$. mp = 88–90 °C. LC-MS (ESI^+^) *m*/*z* = 444.2680 (M + 1), 466.2502 (M + Na). λ_max_ (nm), ε (L·cm^−1^·mol^−1^) = 325 (8157). HPLC purity > 96%.

### 4.4. Free Radical 1,1-Diphenyl-2-picryl-hydrazyl (DPPH)-Scavenging Assay

The total antioxidant capacity of the synthesized compounds was determined using the DPPH radical as a reagent, according to the procedure described by Chang et al. with some modifications [65]. Briefly, 100 μL of 100 μM solution of DDPH in ethanol was added to 10 μL of the synthesized compounds at different concentrations (500, 250, 125, 62.5, 31.25, 15.62, and 7.81 μM). Trolox, a well-known standard with strong antioxidant activities, was used as a positive control; the absorbance was measured at 517 nm after 30 min of incubation at room temperature in the dark using a Tecan Infinite 200 PRO plate reader. The percentage of the inhibition of DPPH oxidation was calculated according to the following formula:

$${{\mathrm{DPPH}}_{\mathrm{scavenging}}}_{\mathrm{effect}\left(\%\right)}=\frac{A_{\mathrm{control}}-A_{\mathrm{sample}}}{A_{\mathrm{control}}}\times 100$$

where $A_{\mathrm{control}}$ means the absorbance of the control sample and $A_{\mathrm{sample}}$ means the absorbance of the standard or tested compound. The results were also expressed as the concentration needed to inhibit a biological process by 50% (IC_50_).

### 4.5. Ferric-Reducing Antioxidant Power (FRAP) Assay

The antioxidant capacity of the synthesized compounds was evaluated by the reduction of the ferric 2,4,6-tripyridyl-s-triazine complex (Fe^3+^-TPTZ) to the ferrous form (Fe^2+^-TPTZ) at low pH, which causes a colored ferrous–tripyridyltriazine complex to form, according to the procedure described by Benzie et al., with some modifications [49]. Briefly, 20 µL of the tested compound was mixed with 130 µL FRAP reagent (buffer acetate pH = 3.6, 10 mM TPTZ, 40 mM HCl, 20 mM FeCl_3_/H_2_O ratio 10:1:1) and then incubated for 45 min at 37 °C. FRAP values were obtained by comparing the absorbance change at 595 nm in test reaction mixtures with those containing ferrous ions in known concentrations using trolox as a calibration curve (concentrations between 7.8 and 500 µM). The results were expressed as μM of trolox equivalent (TE). The absorbance was measured using a Tecan Infinite 200 PRO plate reader.

### 4.6. Inhibition Effects of Compounds ***1***–***9*** on Mushroom TYR Assay

A potassium phosphate buffer (80 μL, 50 mM, pH 6.8), 20 μL of tyrosinase (100 units/mL), and 2 μL of the tested compounds (10–150 μM) dissolved in dimethyl sulfoxide (DMSO) were added to 96-well plates. After incubation at 37 °C for 20 min, 100 μL of L-DOPA (2 mM) was added. The absorbance changes at 475 nm (ε = 3.7 × 10^3^ M^−1^cm^−1^) were immediately monitored for 20 min at 37 °C using a Tecan Infinite 200 PRO plate reader. Kojic acid, a well-established tyrosinase inhibitor, served as a positive control. Dose–response curves were generated by conducting assays with increasing concentrations of each inhibitor. To ensure measurement accuracy, all experiments were performed in triplicate, with the average value used as the final result. The inhibitory potency of each compound was expressed as the IC_50_ value, representing the concentration required to reduce enzyme activity by 50%, which was determined by interpolating the dose–response curves.

For monophenolase inhibition assays, a potassium phosphate buffer (80 μL, 50 mM, pH 6.8), 20 μL of tyrosinase (100 units/mL), and 2 μL of the tested compounds (30 μM) dissolved in DMSO were added to 96-well plates. After incubation at 37 °C for 20 min, 100 μL of L-tyrosine (2 mM) was added. The absorbance changes at 475 nm were immediately monitored for 40 min at 37 °C using a Tecan Infinite 200 PRO plate reader. The lag time was determined from the plot of absorbance at 475 nm versus time (min) by extrapolating the linear portion of the curve to the *x*-axis.

### 4.7. Inhibition Kinetics of Compound ***5***

To evaluate the inhibition kinetics of tyrosinase, assays were conducted in phosphate buffer (50 mM, pH 6.8) using L-DOPA as the substrate (0.1–1 mM) and tyrosinase (100 units/min) in the presence of varying concentrations of compound **5** (2.5–30 μM) under the same conditions as described in Section 4.4. Absorbance changes at 475 nm were continuously monitored for 40 min at 37 °C using a Tecan Infinite 200 PRO plate reader. Initial reaction velocities (V_0_) were determined for each inhibitor concentration, and kinetic parameters (Kₘ and Vₘₐₓ) were analyzed using Lineweaver–Burk and Michaelis–Menten plots to determine the type of inhibition [66,67,68]. All experiments were performed in triplicate to ensure reproducibility.

### 4.8. Fluorescence Quenching Assay

The fluorescence quenching spectra were recorded with an excitation wavelength (λ_ex_ = 280 nm) and excitation and emission slit widths of 5 nm. A potassium phosphate buffer (80 μL, 50 mM, pH 6.8), 20 μL of tyrosinase (100 units/mL), and 2 μL of the tested compounds (10–100 μM) dissolved in DMSO were added to 96-well plates. After incubating at 37 °C for 20 min, the fluorescence spectrum was recorded at an emission range from 315 to 415 nm. Fluorescence quenching is described by the Stern–Volmer equation [69]:

$$\frac{F_{0}}{F}=1+K_{q}\tau_{0}[Q]=1+k_{\mathrm{sv}}[Q]$$

For the static quenching interaction, the binding affinity (K_a_) and the number of binding sites (n) between the inhibitor and TYR were determined using the following calculations [70,71]:

(1) $$\log\left(\frac{F_{0}-F}{F}\right)=\mathrm{nlog}\left[Q\right]+\log k_{a}$$

where F and F_0_ represent the fluorescence intensities of protein in the presence and absence of a quencher, respectively. [Q] is the quencher concentration. K_sv_ is the Stern–Volmer quenching constant, and k_q_ is the rate constant of the bimolecular quenching process. τ_0_ is the average fluorescence lifetime of the fluorophores in the absence of the quencher, with a typical value of 10^−8^ s for biological macromolecules [72].

### 4.9. Interaction with Copper Ions

To a 96-well plate, 2 μL of the CuSO_4_ solution (1 mM), 2 μL of the tested compounds (1 mM), and 96 μL of PBS were added. After incubation for 5 min, the UV-Vis spectra were recorded in the range of 280–500 nm and compared to those of the free compounds.

### 4.10. Statistical Analysis

Data are expressed as mean ± SD. Statistical analysis was performed using a one-way analysis of variance. Linear regression was performed to identify a possible dose-dependent effect. Values of *p* < 0.05 were considered significant.

## 5. Conclusions

In conclusion, in this study, we have successfully synthesized and characterized novel thiosemicarbazone–CBD–aldehyde derivatives (**5**–**9**), extending our previous work on compounds (**1**–**4**) [37]. These derivatives demonstrated notable antioxidant activity and meaningful inhibitory effects on both the monophenolase and diphenolase activity of mushroomTYR, with compound **5** exhibiting the highest potency. The observed extension of the lag phase, particularly with compound **5**, suggests a potential mechanism involving the reduction of copper ions at the enzyme’s active site, likely mediated by the thiosemicarbazone moiety. SAR analysis revealed the crucial role of NH_2_ and OH groups in modulating tyrosinase inhibition. Modifications to the NH_2_ group had a substantial impact, with methyl and dimethyl substitutions slightly reducing activity, while the phenyl group led to a more pronounced decrease, likely due to steric hindrance. Similarly, derivatization of the OH groups on the CBD skeleton affected activity in a substrate-dependent manner, maintaining or enhancing inhibition against L-DOPA but reducing it when L-tyrosine was the substrate. While these findings indicate the potential of these derivatives as TYR modulators, further studies are necessary to gain deeper insights into their precise mechanisms of action.

Beyond their role as tyrosinase inhibitors, these CBD-derived thiosemicarbazones may offer additional therapeutic benefits due to their anti-inflammatory properties. Since hyperpigmentation disorders are often linked to oxidative stress and inflammation, these compounds may serve a dual function, not only inhibiting TYR activity but also mitigating the underlying inflammatory processes. This expands their potential application in both therapeutic and cosmetic formulations, making them promising candidates for addressing pigmentation-related conditions.

Ongoing studies in our laboratory are further exploring the biological activity of these derivatives. Investigations are underway to assess their anti-inflammatory effects in cell culture models, which will help to determine their broader pharmacological potential. Moreover, we are examining their impact on the ECS and the possible interplay between ECS signaling and tyrosinase regulation, which could reveal novel insights into their multifunctional biological roles.

This integrated approach aims to enhance our understanding of CBD–thiosemicarbazone derivatives, paving the way for their further optimization and potential development as therapeutic agents for tyrosinase-related disorders. While this study represents a preliminary investigation, it lays the foundation for continued research into the diverse pharmacological properties of these promising compounds.

## Figures and Tables

**Table 1:** IC_50_ values of compounds **1**–**9** against mushroom TYR catalyzing oxidation of L-DOPA.

Compound	IC_50_ [μM]
**1**	>100
**2**	>100
**3**	>100
**4**	>100
**5**	27.72 ± 1.84
**6**	29.38 ± 2.22
**7**	28.07 ± 1.82
**8**	42.16 ± 3.52
**9**	22.41 ± 2.90
KA	35.33 ± 1.54

**Table 2:** Effect of compounds **5**–**9** (20 μM) on the lag time values of the oxidation step of L-tyrosine by mushroom TYR.

Compound	Lag Time [min]
without inhibitor	4.40 ± 0.04
**5**	9.89 ± 0.66
**6**	6.06 ± 0.11
**7**	6.19 ± 0.27
**8**	4.58 ± 0.04
**9**	6.67 ± 0.17
KA	11.31 ± 0.05

**Table 3:** Calculated fluorescence quenching parameters of TYR with compounds **5**–**9** and binding constant and site number of TYR with each of thiosemicarbazone–CBD–aldehyde derivatives.

Compound	K_sv_ (M^−1^)	k_q_ (M^−1^·s^−1^)	k_a_ (M^−1^)	n
**5**	7.61 × 10^3^	7.61 × 10^11^	1.21 × 10^4^	1.05
**6**	9.98 × 10^3^	9.98 × 10^11^	0.87 × 10^4^	1.08
**7**	1.38 × 10^4^	1.38 × 10^12^	0.52 × 10^4^	0.92
**8**	4.68 × 10^4^	4.68 × 10^12^	2.55 × 10^4^	1.19
**9**	1.30 × 10^4^	1.30 × 10^12^	1.31 × 10^4^	1.01

## Data Availability

Data is contained within the article and Supplementary Materials.

## Supplementary Materials

The following supporting information can be downloaded at https://www.mdpi.com/article/10.3390/molecules30061291/s1: Figure S1. GC-MS chromatogram of 3-methyl cannabidiol dimethyl ether. Figure S2. NMR spectra for compounds **5**–**9**. Figure S3. FTIR spectra of compounds **5**–**9**. Figure S4. ESI-MS spectra of compounds **5**–**9**. Figure S5. Fluorescence quenching spectra and Stern–Volmer plots for compounds **6**–**9**. Figure S6. UV-Vis spectra of free and copper complexes of compounds **5**–**9.** Figure S7. ESI-MS spectra of copper complexes of compounds **6**–**9**.
