# Supplementary material for: Cannabidiol-Based Thiosemicarbazones: A Preliminary Study Evaluating Their Anti-Tyrosinase Properties
Source: Molecules. 2025 Mar 13;30(6):1291. doi: 10.3390/molecules30061291 (PMC11944387; doi:10.3390/molecules30061291)
Supplement: Supplementary file 1 [file molecules-30-01291-s001.zip › molecules-3486160-supplementary.pdf]

Supporting information

Cannabidiol-Based Thiosemicarbazones: A Preliminary Study Evaluating Their Anti-tyrosinase properties

Eliav Peretz [1,2], Noa Ashkenazi [1] and Sanaa Musa [1,2*]

1 Department of Biotechnology, Tel-Hai Academic College, Kiryat Shmona 11016, Israel
2 Natural Compounds and Organic Synthesis Laboratory, Migal-Galilee Research Institute, Kiryat Shmona 11016, Israel
* Correspondence: sanaa@migal.org.il musasan@telhai.ac.il;

General Considerations. All chemicals and reagents were purchased from Sigma-Aldrich. Anhydrous tetrahydrofuran was dried over sodium and used freshly. HPLC-grade acetonitrile and formic acid were used. Flash column chromatography was performed with Merck ultra-pure silica gel (230-400 mesh). Yields refer to isolated compounds greater than 95% purity as determined by proton Nuclear Magnetic Resonance spectroscopy (^{1}H-NMR), High-performance liquid chromatography (HPLC), and Gas chromatography (GC) analysis.

HPLC Analysis

HPLC analysis was performed with UHPLC connected to a photodiode array detector (Agilent 1290), with a reverse-phase column (Phenomenex RP-18, 150 X 4.6 mm, 3 µm). The mobile phase was a mixture of (A) double-distilled water (DDW) with 0.1% formic acid and (B) acetonitrile with 0.1% formic acid with a flow of 0.5ml/min and gradient starting with 5% B and increasing in a concentration to 95% B for 30 min and then kept at 95% B for an additional 5 min.

LC–MS Analysis

The LC-MS analysis was performed with a heated electrospray ionization (ESI) source connected to a waters ZQ mass detector. The ESI capillary voltage was set to 3900 V, capillary temperature to 250°C, and gas temperature to 350°C. Nitrogen gas (N_2) was used. The MS conditions were set as follows: the flow rate of sheath gas, aux gas, and sweep gas was kept at 35 L/min, 10 L/min, and 1 L/min, respectively.

GC-MS analysis

The analysis of the compounds was performed using gas chromatogram instrument (Agilent 7890A), equipped with Phenomenex Zebron ZB-5 column (30m, 0.32mm, 0.25µm) coupled with mass spectrometer (Agilent 5975C). The injection was made in split mode (10:1) with an injection volume of 1µl. The injector temperature was set to 240 °C. Helium was used as a carrier gas at a constant flow rate of 1 ml/min. The initial oven

temperature was held at 50 °C for 5 min, then increased to 300 °C at a rate of 10 °C/min, and finally held at this temperature for 5 min (total time 35 min)

NMR analysis:

The synthesized compounds were dissolved in deuterated chloroform or dimethyl sulfoxide ($CDCl_3$ or $(CD_3)_2SO$). ^1H- NMR spectra were recorded at room temperature with a Bruker 400 MHz instrument, with chemical shifts reported in ppm relative to the residual deuterated solvent.

FTIR and UV spectroscopic analysis:

FTIR spectra were recorded in the Nicolet™ iS™ 10 FTIR Spectrometer (Thermo Scientific™). UV spectra were recorded by Tecan Infinite 200 PRO spectrophotometer.

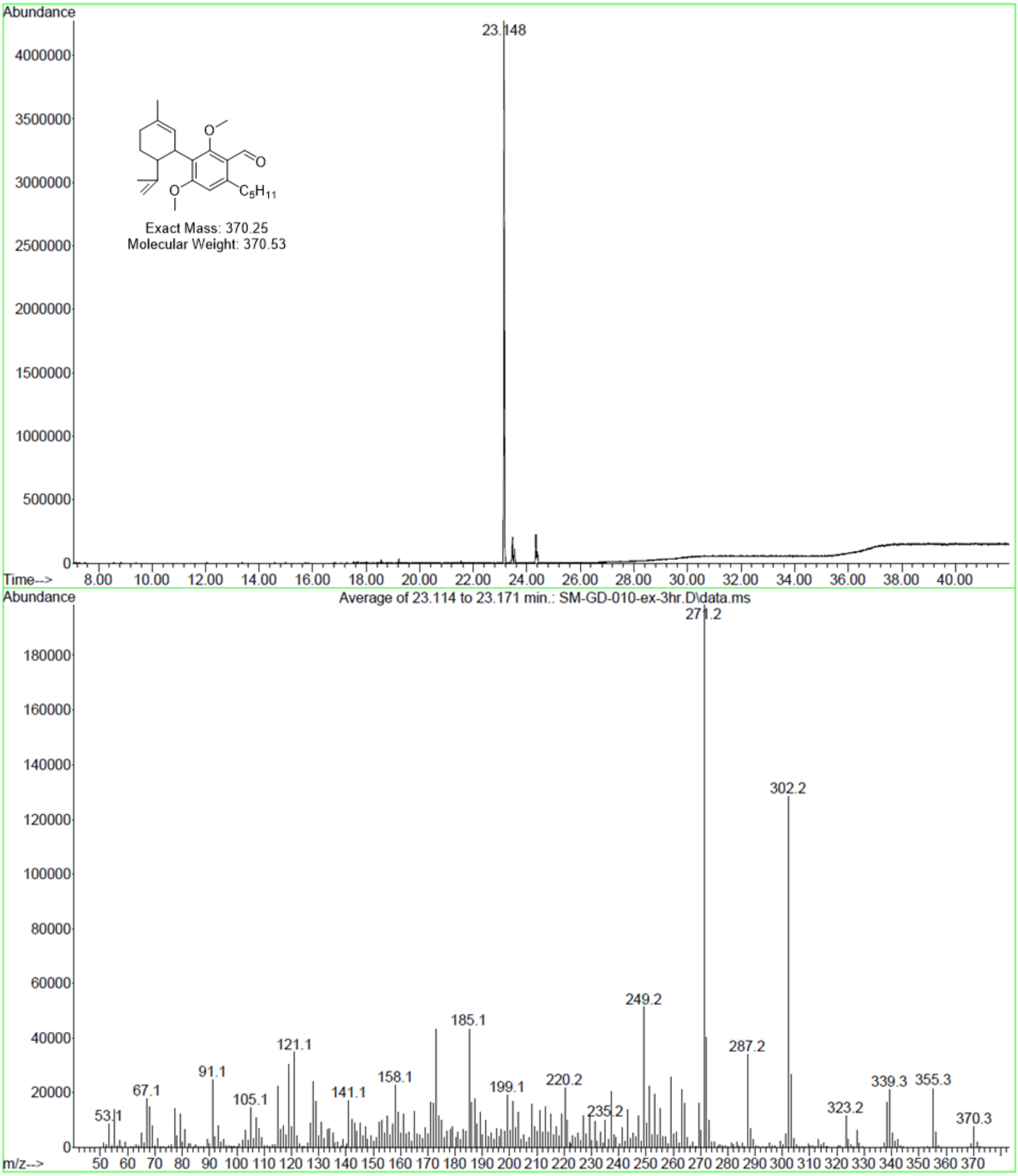

Figure S1. GCMS of compound 3-formyl cannabidiol dimethyl ether

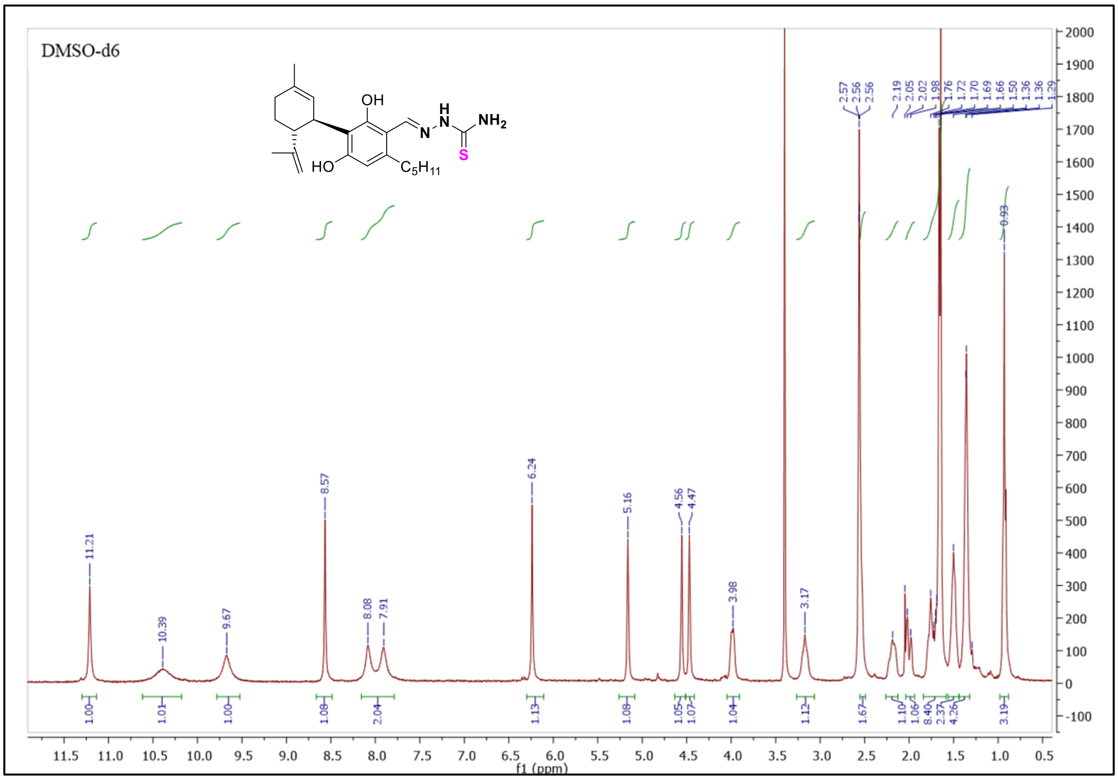
DMSO-d6
OH
H
N
NH2
S
HO
C5H11
f1 (ppm)

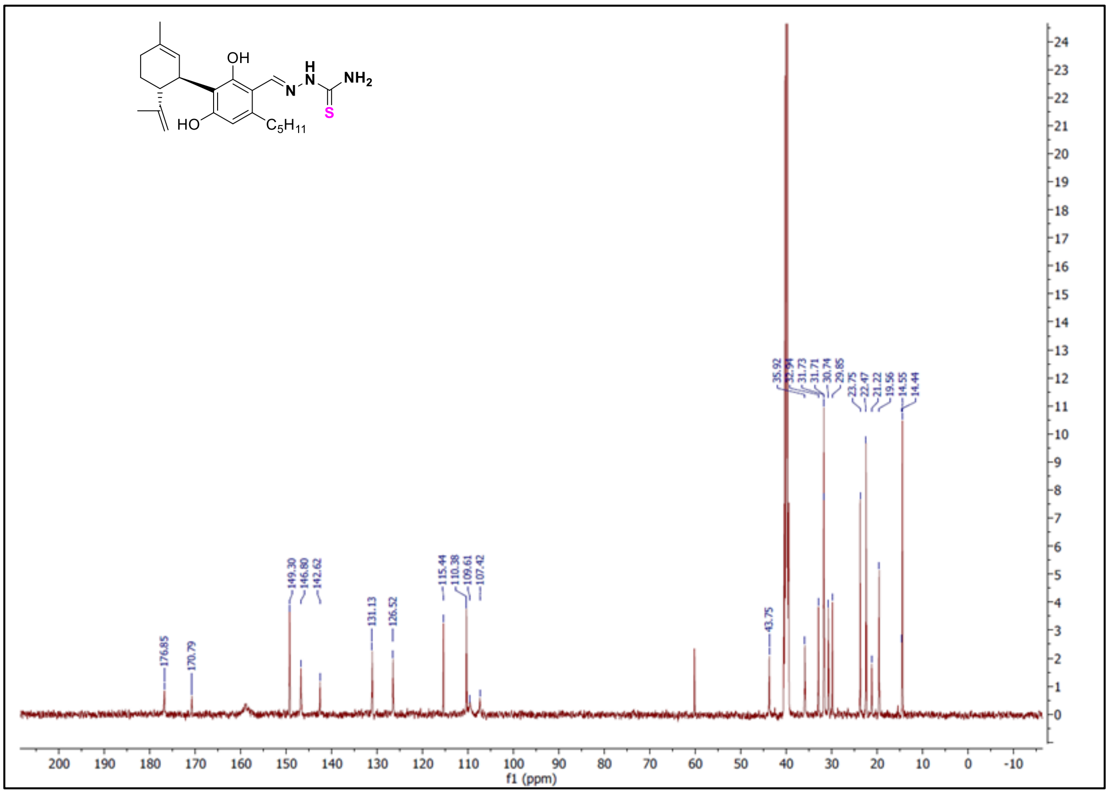
OH
H
N
NH2
S
HO
C5H11
f1 (ppm)

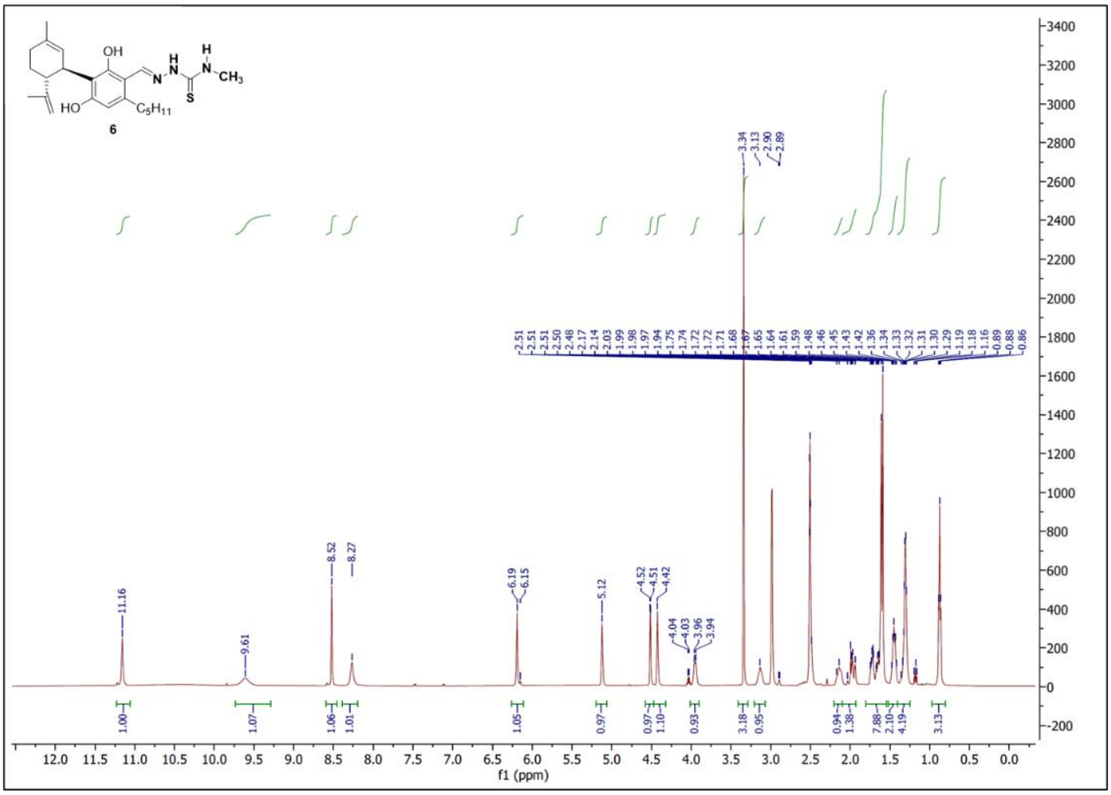

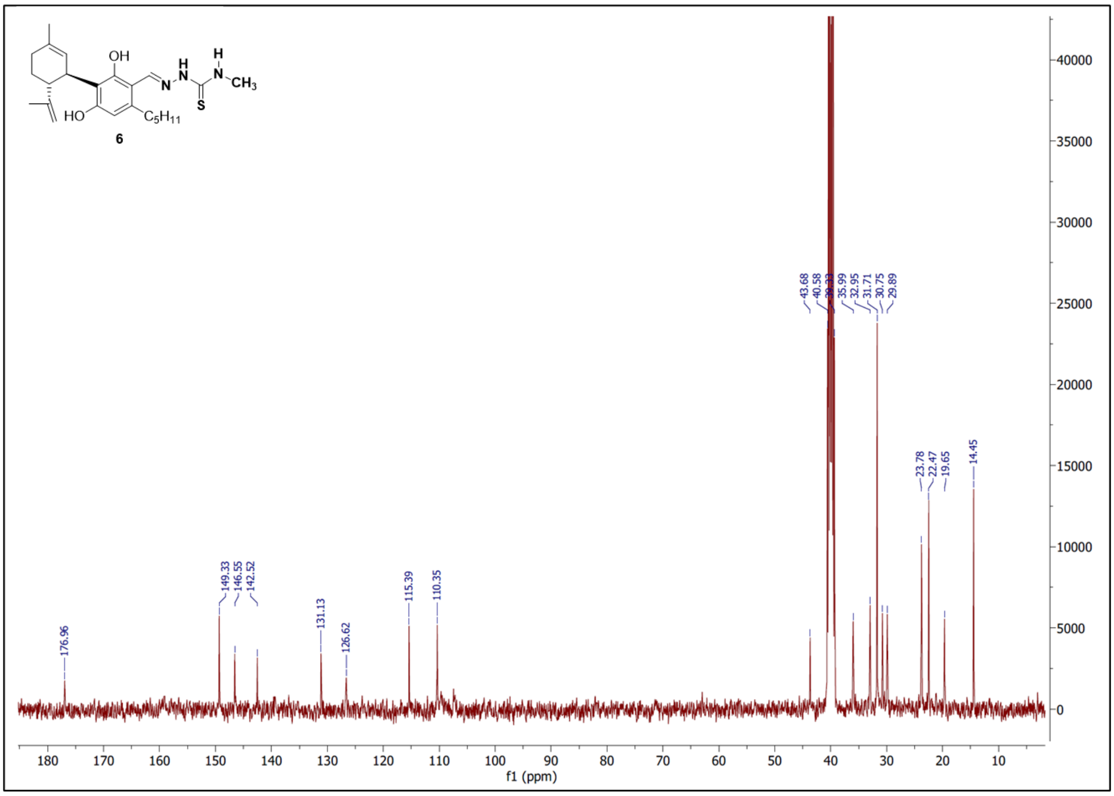

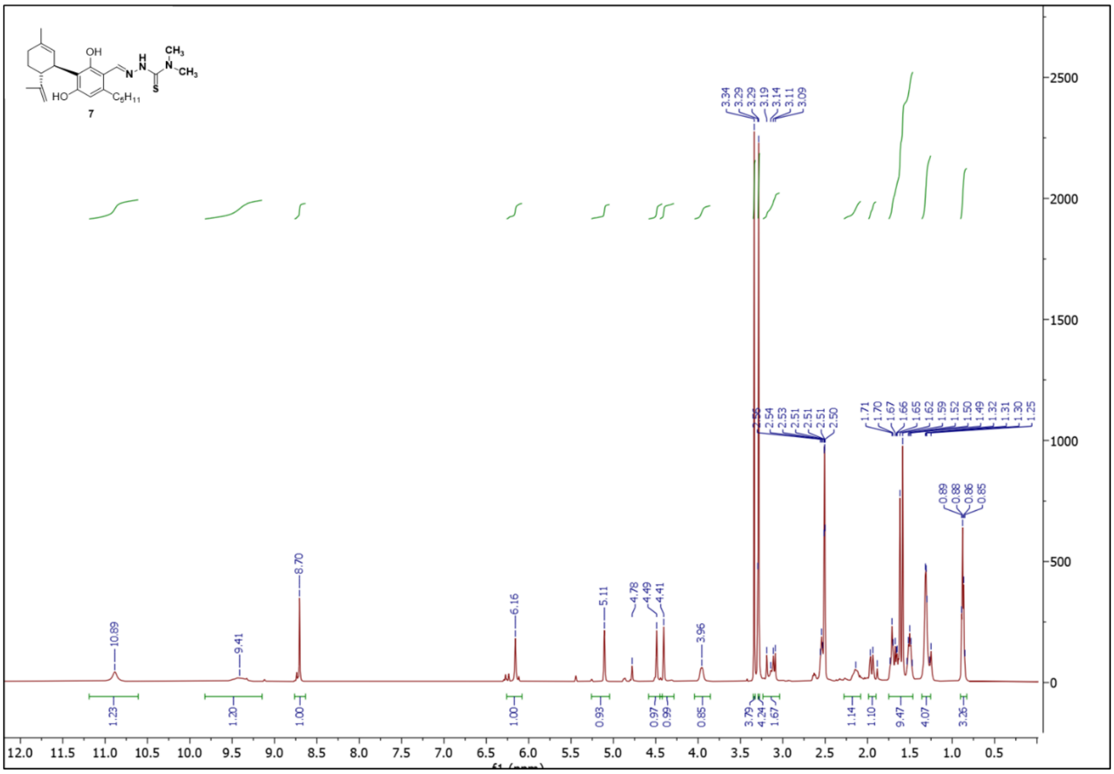
7
f1 (ppm)

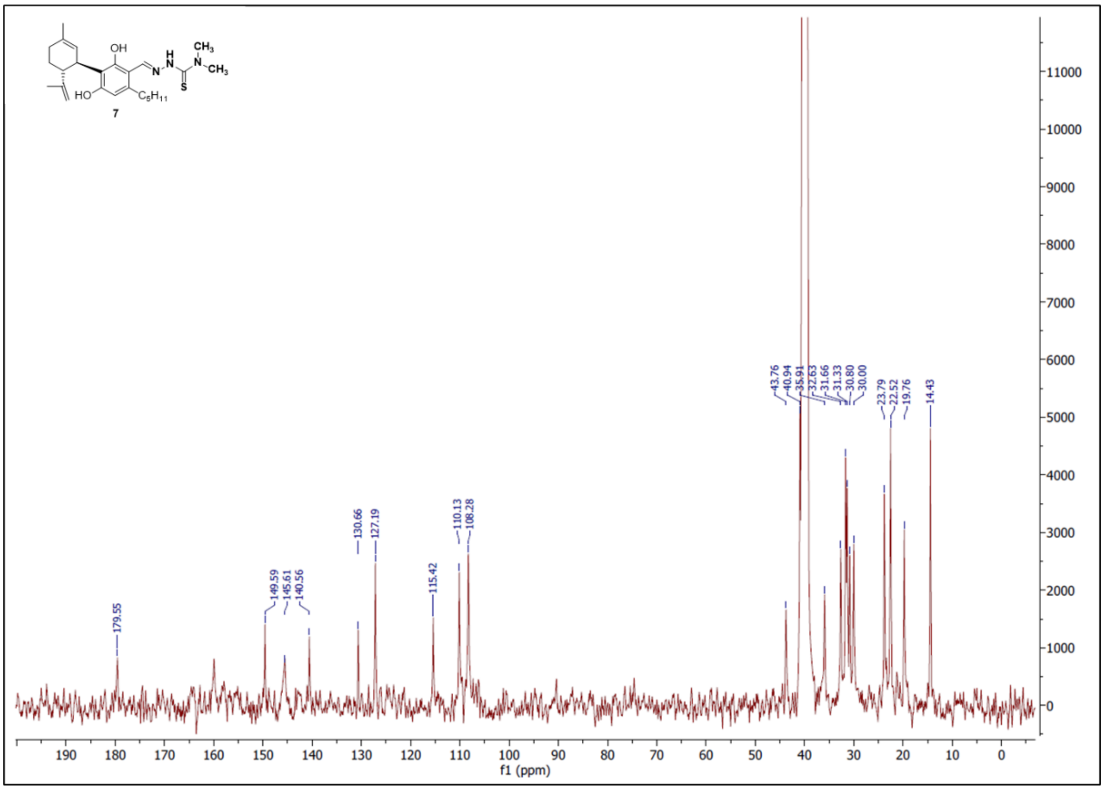
7
f1 (ppm)

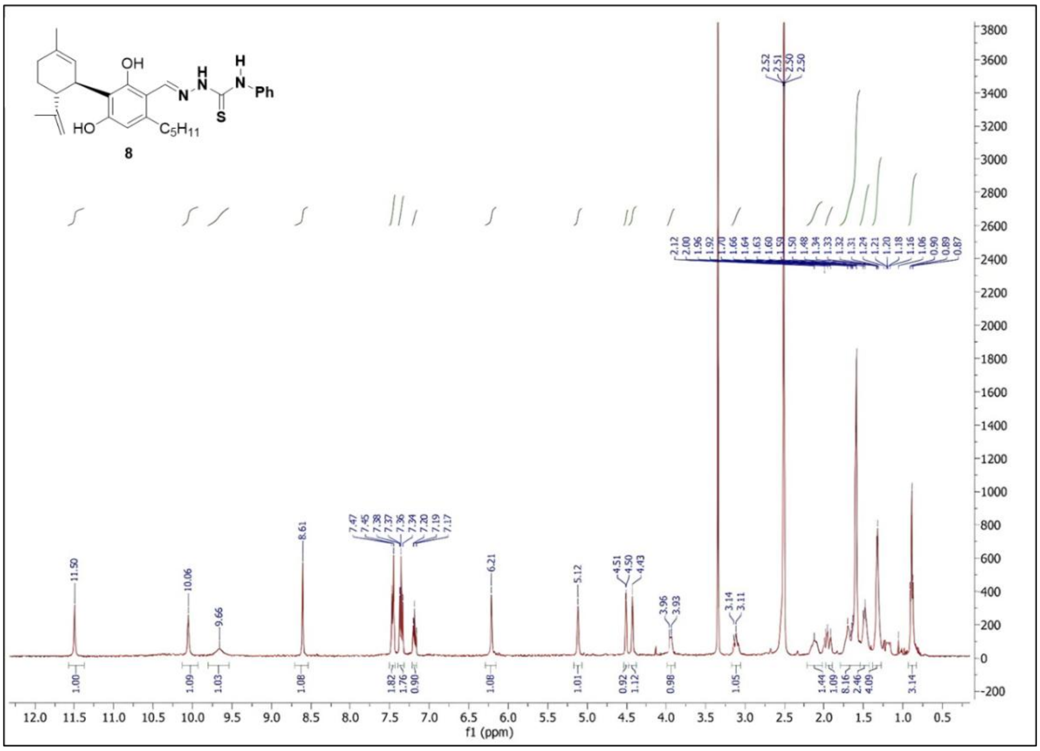

OH
H
N
N
H
N
Ph
S
HO
C5H11
8
11.50
10.06
9.66
8.61
7.47
7.45
7.38
7.37
7.36
7.34
7.20
7.19
7.17
6.21
5.12
4.51
4.50
4.43
3.96
3.93
3.14
3.11
2.52
2.51
2.50
2.50
1.00
1.09
1.03
1.08
1.82
1.76
0.90
1.08
1.01
0.92
1.12
0.98
1.05
1.44
1.09
8.16
2.46
4.09
3.14
f1 (ppm)

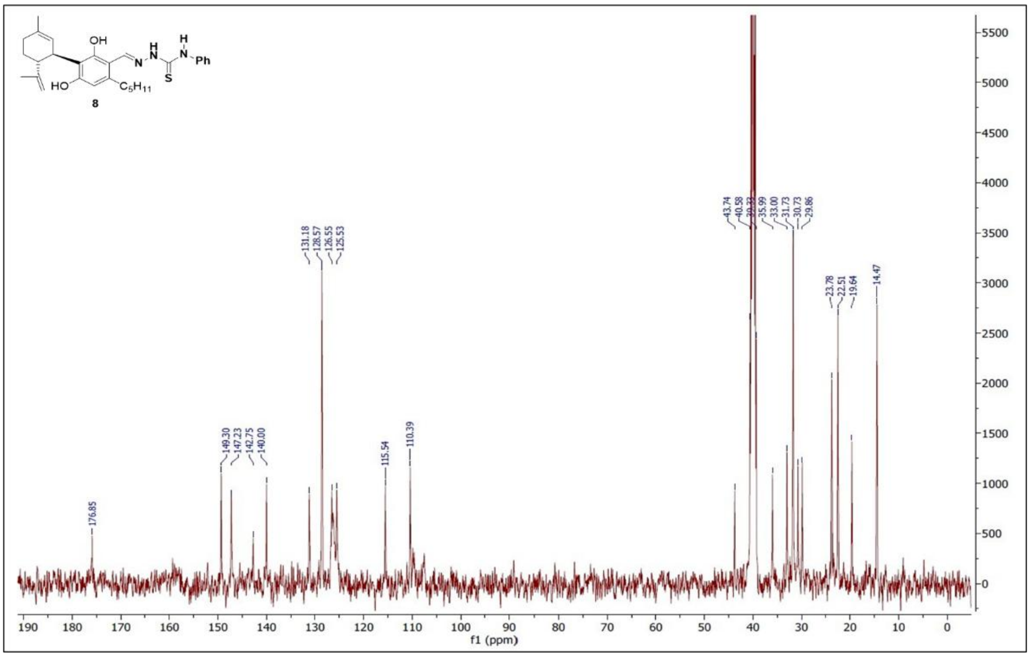

OH
H
N
N
H
N
Ph
S
HO
C5H11
8
176.85
149.30
147.23
142.75
140.00
131.18
128.57
126.55
125.53
115.54
110.39
43.74
40.58
35.59
33.00
31.73
30.73
29.86
23.78
22.51
19.64
14.47
f1 (ppm)

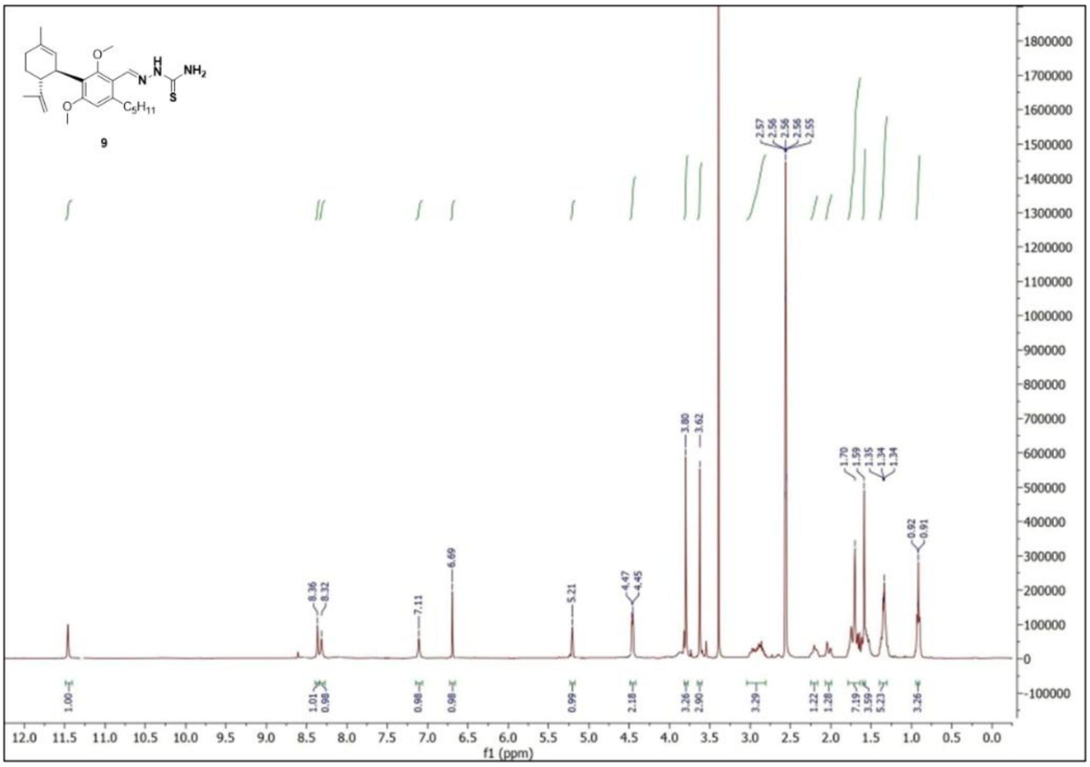

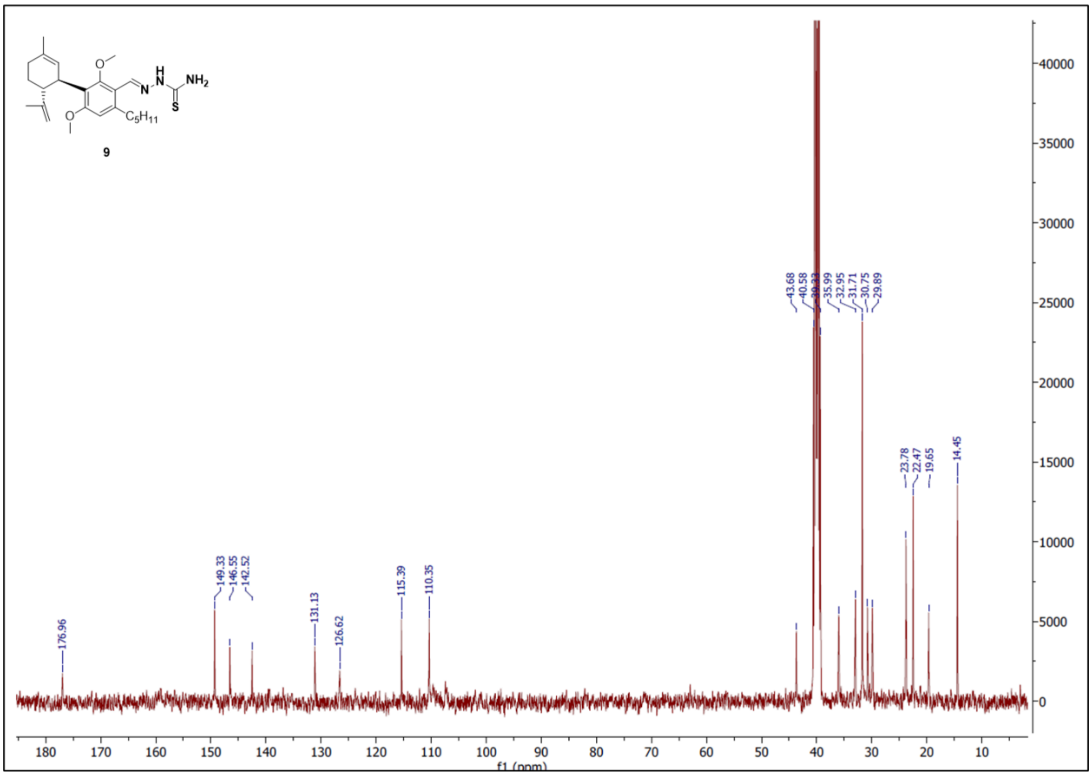

Figure S2. ^{1}H- and ^{13}C-NMR spectra of compound **5-9**, 400 MHz. DMSO-d_6 was used as the deuterated solvent.

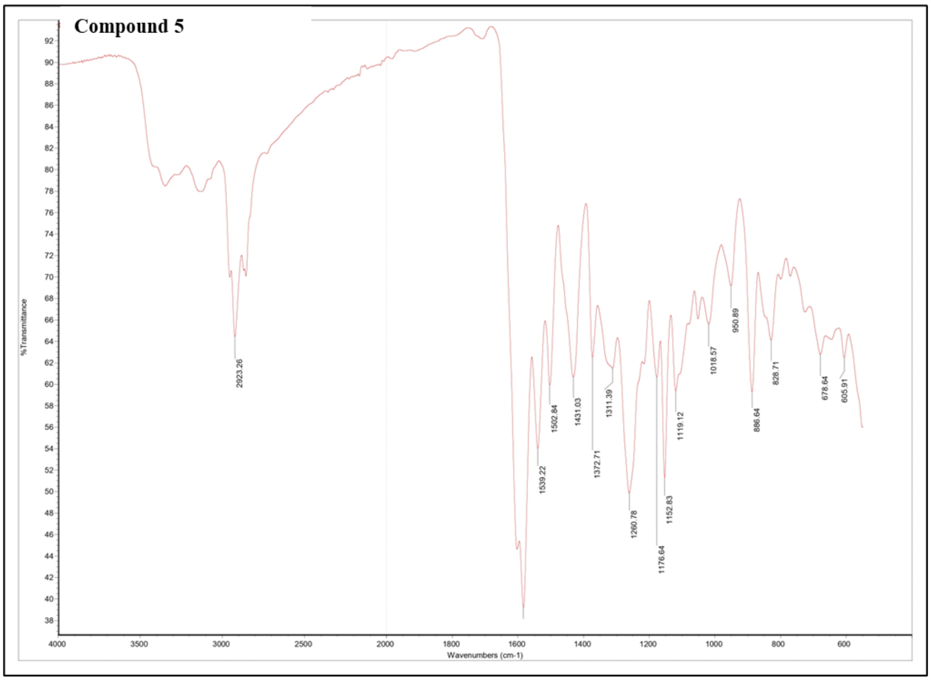
Compound 5
%Transmittance
Wavenumbers (cm-1)
2923.26
1539.22
1502.84
1431.03
1372.71
1311.39
1260.78
1176.64
1152.83
1119.12
1018.57
950.89
886.64
828.71
678.64
605.91

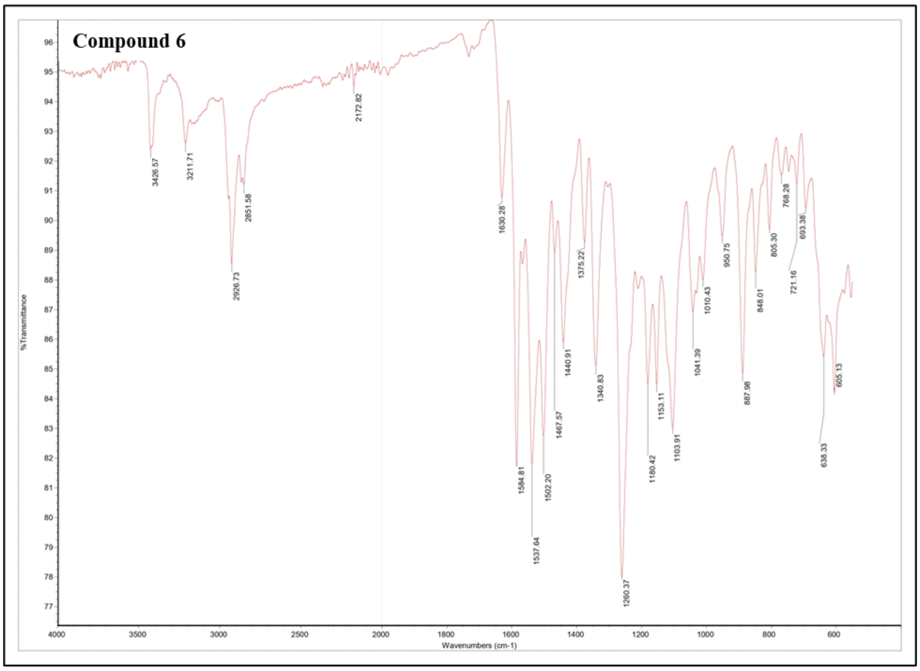
Compound 6
%Transmittance
Wavenumbers (cm-1)
3426.57
3211.71
2926.73
2851.58
2172.82
1630.28
1584.81
1537.64
1502.20
1467.57
1440.91
1375.22
1340.83
1260.37
1180.42
1153.11
1103.91
1041.39
1010.43
950.75
887.98
848.01
805.30
768.28
721.16
693.38
638.33
605.13

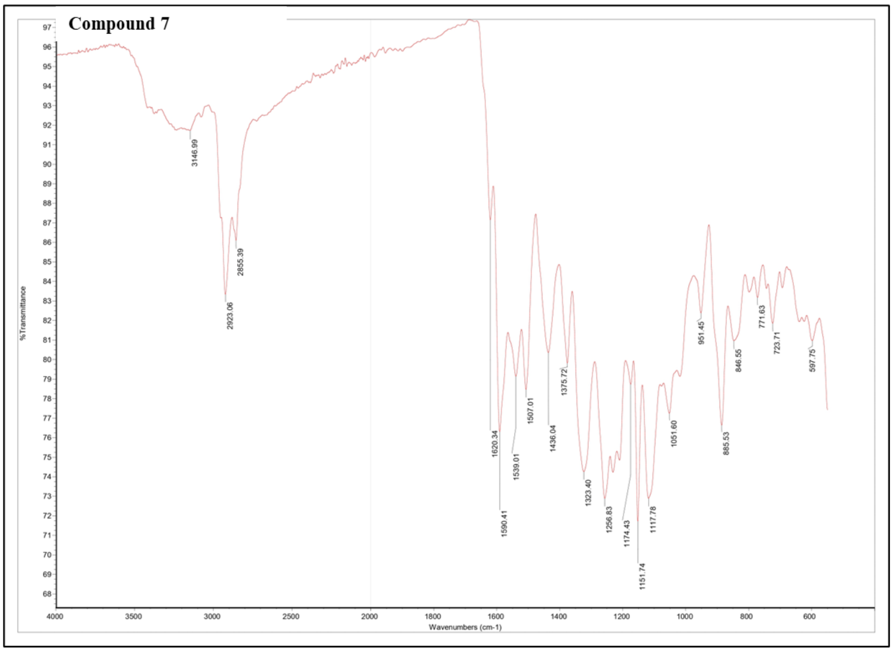
Compound 7
%Transmittance
Wavenumbers (cm-1)
3146.99
2923.06
2855.39
1620.34
1590.41
1539.01
1507.01
1436.04
1375.72
1323.40
1256.83
1174.43
1151.74
1117.78
1051.60
951.45
885.53
846.55
771.63
723.71
597.75

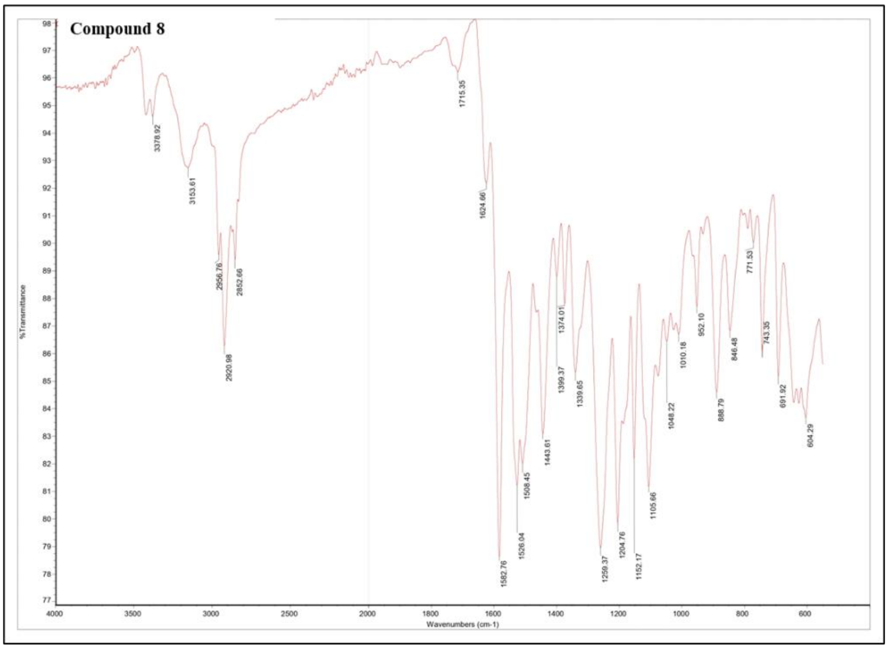
Compound 8
%Transmittance
Wavenumbers (cm-1)
3378.92
3153.61
2956.76
2920.98
2852.66
1715.35
1624.66
1582.76
1526.04
1508.45
1443.61
1399.37
1374.01
1339.65
1259.37
1204.76
1152.17
1105.66
1048.22
1010.18
952.10
888.79
846.48
771.53
743.35
691.92
604.29

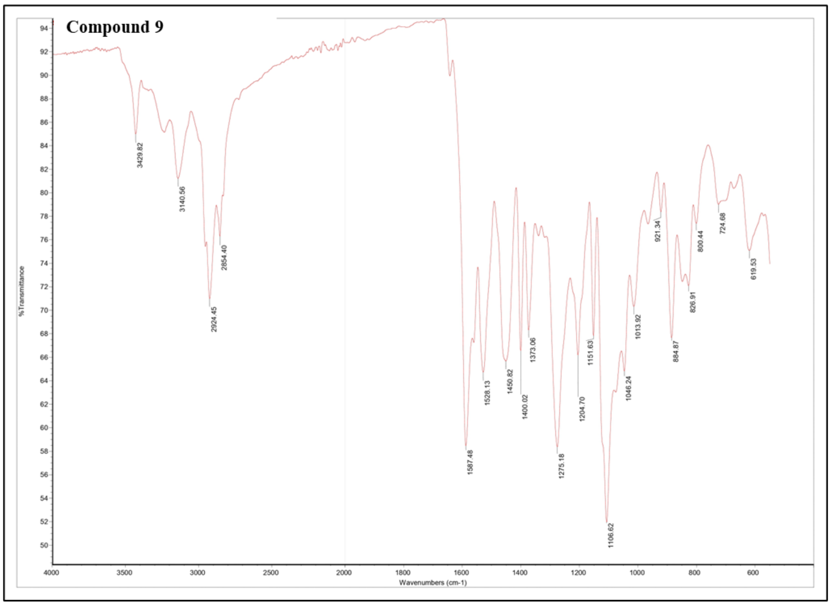

Figure S3. FTIR spectra of compounds **5-9**.

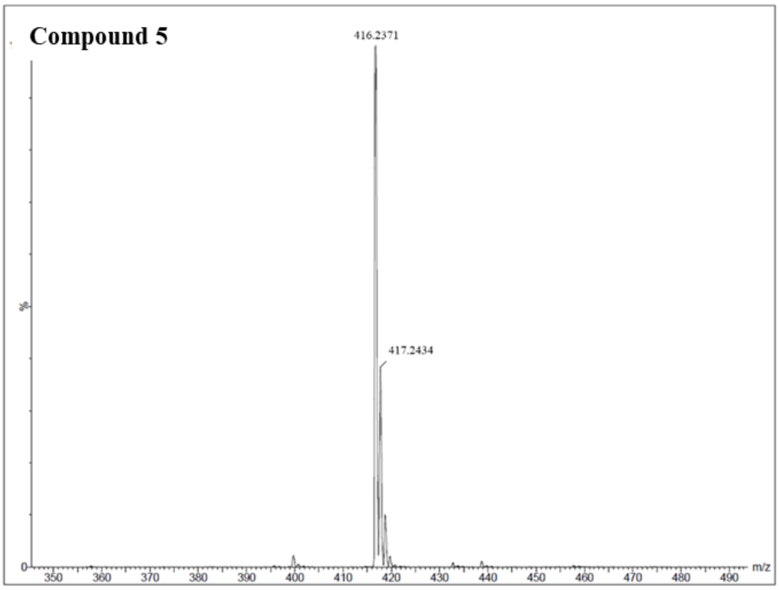
Compound 5
416.2371
417.2434
%
0
350
360
370
380
390
400
410
420
430
440
450
460
470
480
490
m/z

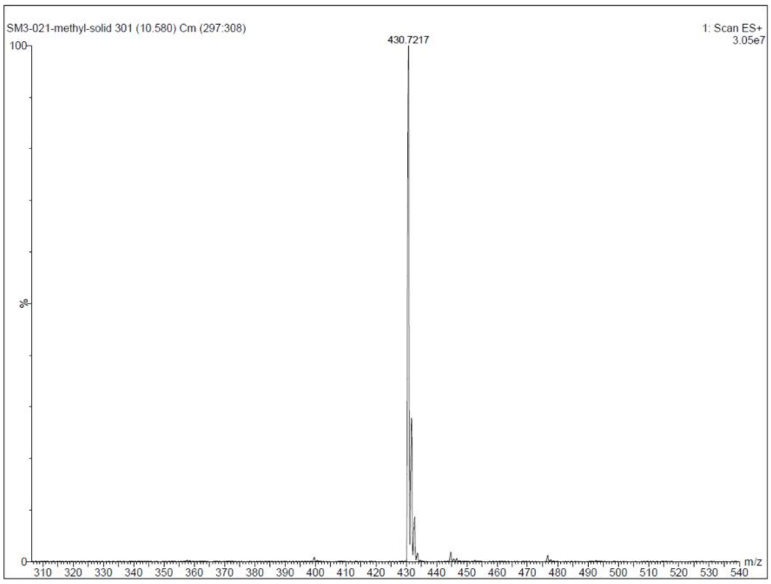
SM3-021-methyl-solid 301 (10.580) Cm (297:308)
1: Scan ES+
3.05e7
430.7217
100
%
0
310
320
330
340
350
360
370
380
390
400
410
420
430
440
450
460
470
480
490
500
510
520
530
540
m/z

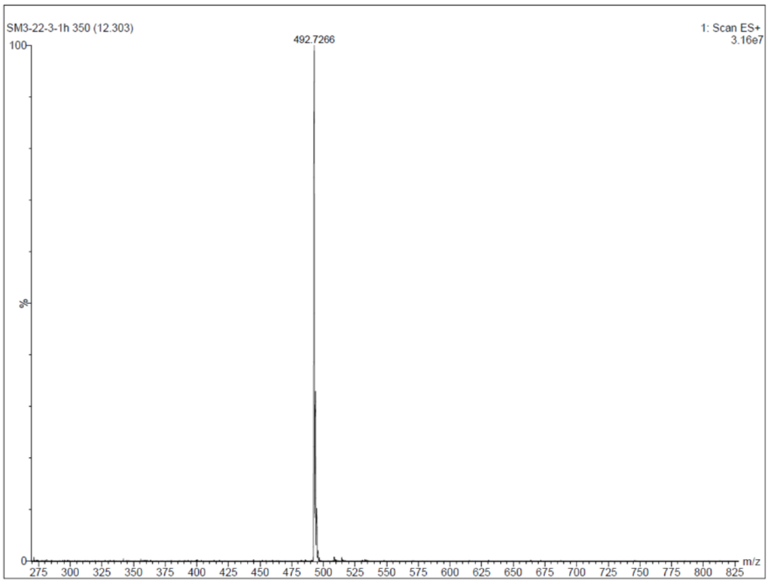

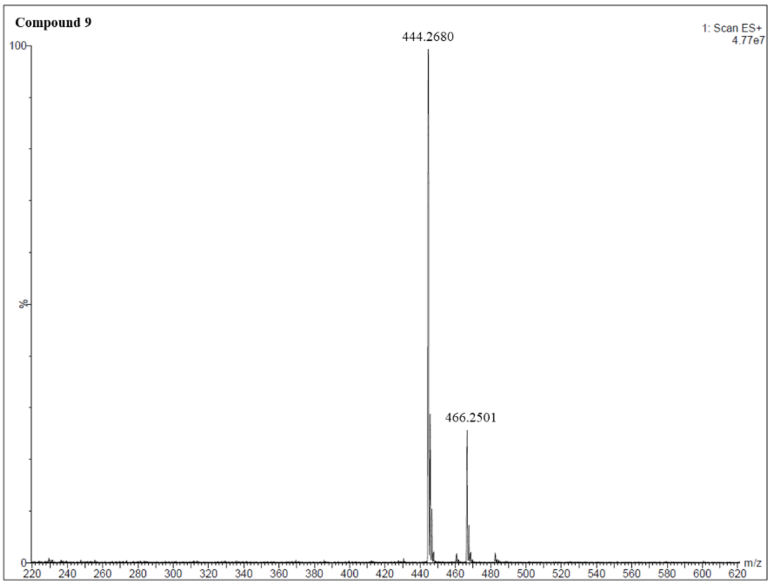

Figure S4. ESI-MS spectra of compounds **5-9.**

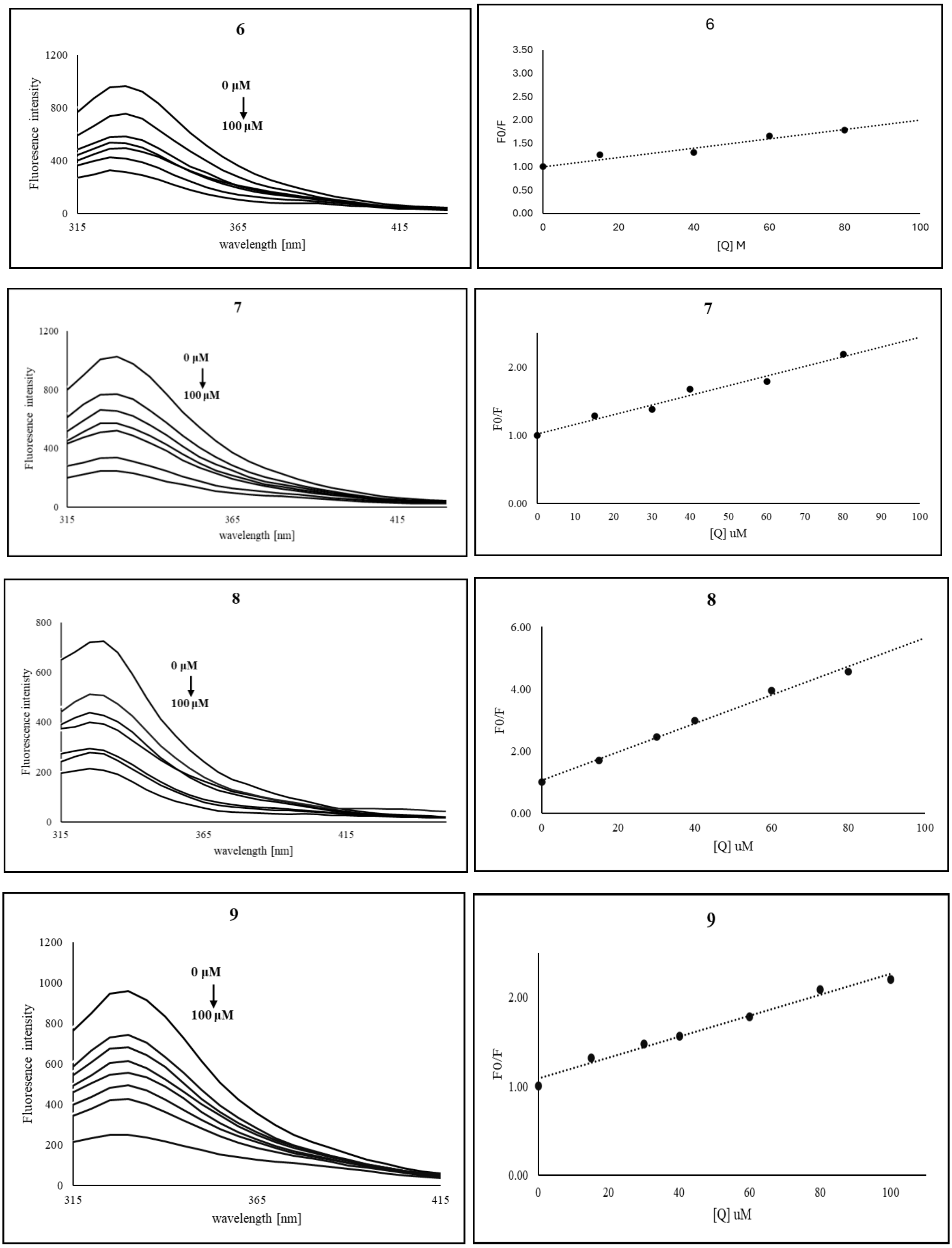

Figure S5. (left) Fluorescence quenching of TYR at λ_{ex}=280 nm in increasing concentrations of **6-9** at 310 K. (right) Stern-Volmer plots of each tested compound.

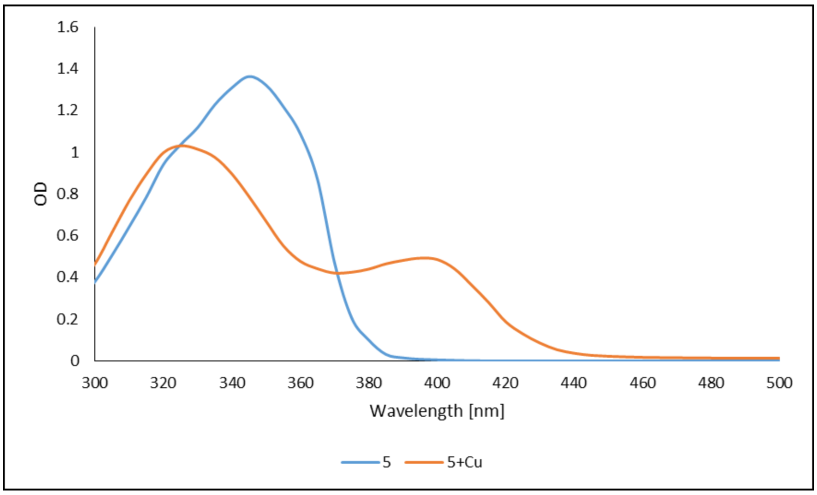
OD
1.6
1.4
1.2
1
0.8
0.6
0.4
0.2
0
300
320
340
360
380
400
420
440
460
480
500
Wavelength [nm]
5
5+Cu

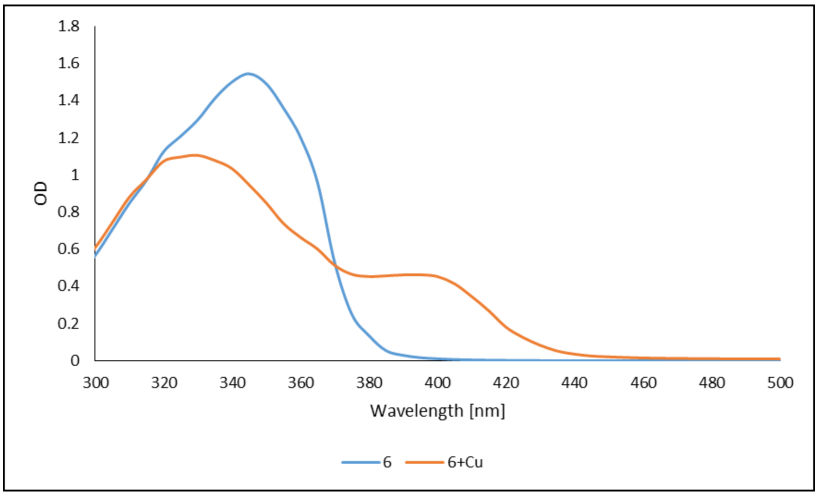
OD
1.8
1.6
1.4
1.2
1
0.8
0.6
0.4
0.2
0
300
320
340
360
380
400
420
440
460
480
500
Wavelength [nm]
6
6+Cu

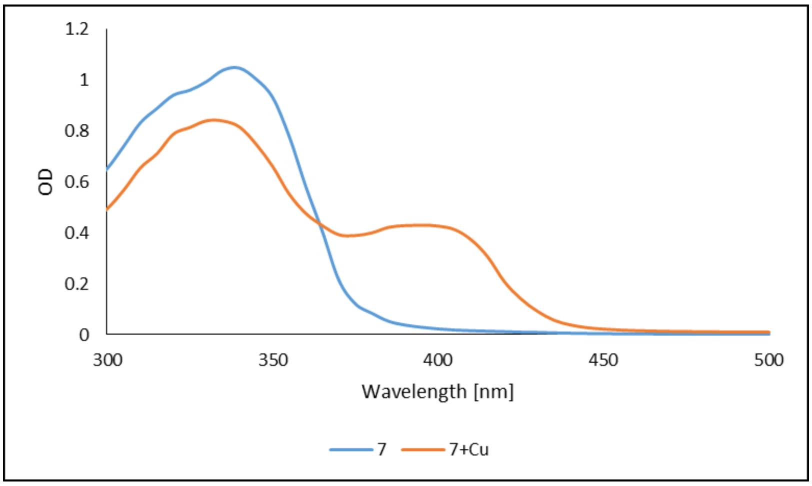
OD
1.2
1
0.8
0.6
0.4
0.2
0
300
350
400
450
500
Wavelength [nm]
7
7+Cu

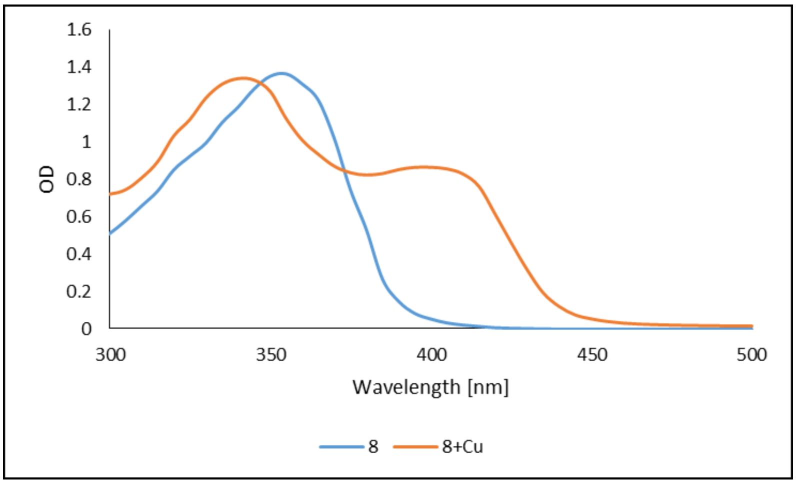

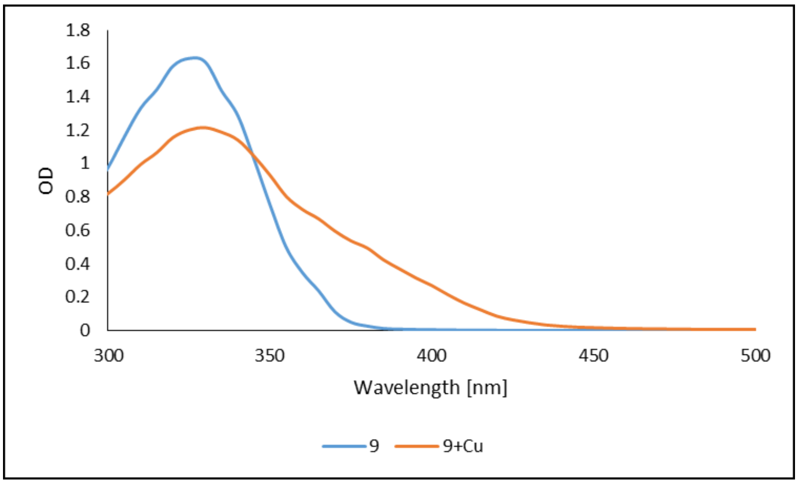

Figure S6. UV-Vis spectra of compounds **5-9** (blue line) and their copper complexes (orange line).

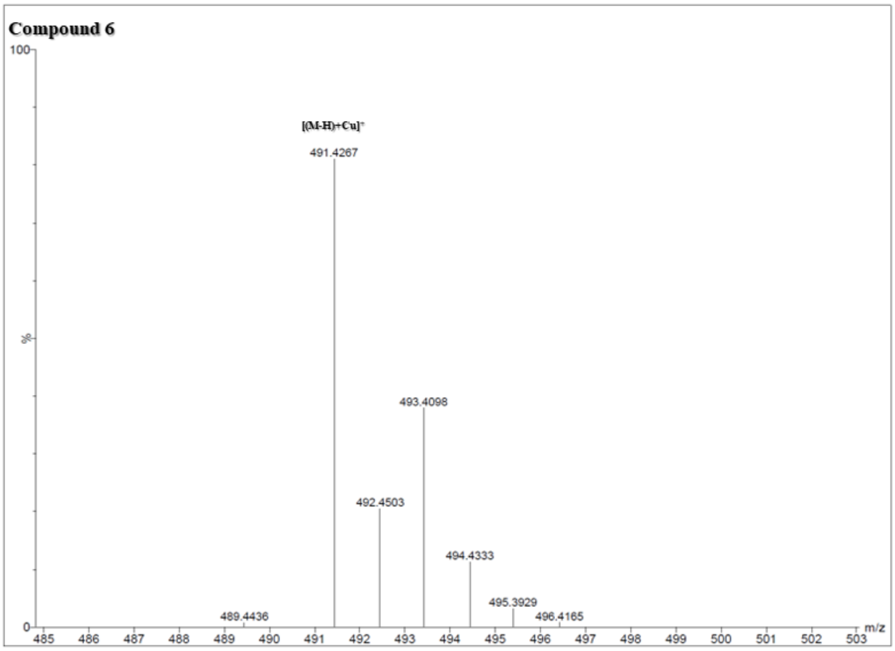
Compound 6
100
[(M-H)+Cu]+
491.4267
%
493.4098
492.4503
494.4333
495.3929
489.4436
496.4165
0
485 486 487 488 489 490 491 492 493 494 495 496 497 498 499 500 501 502 503
m/z

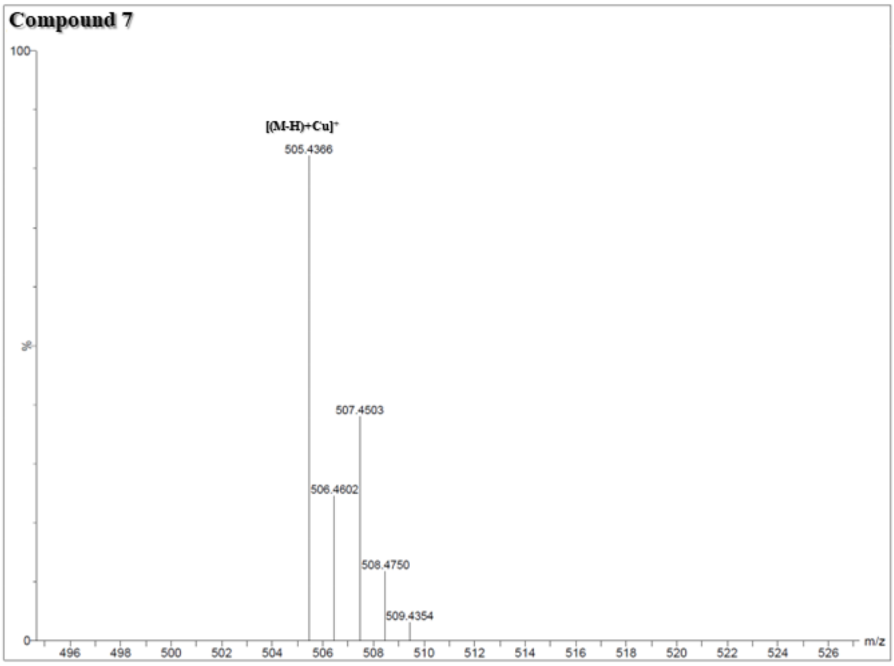
Compound 7
100
[(M-H)+Cu]+
505.4366
%
507.4503
506.4602
508.4750
509.4354
0
496 498 500 502 504 506 508 510 512 514 516 518 520 522 524 526
m/z

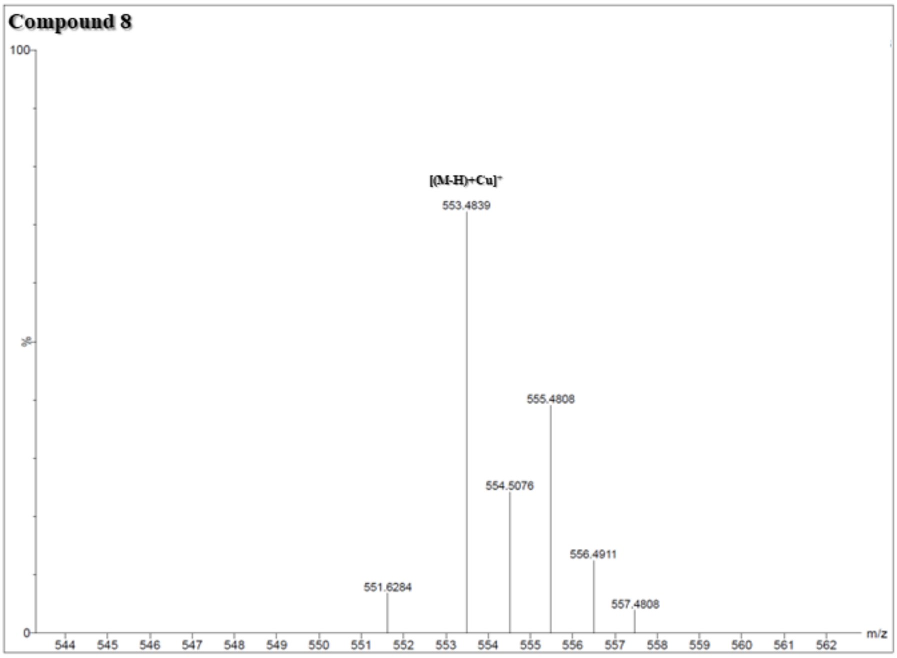

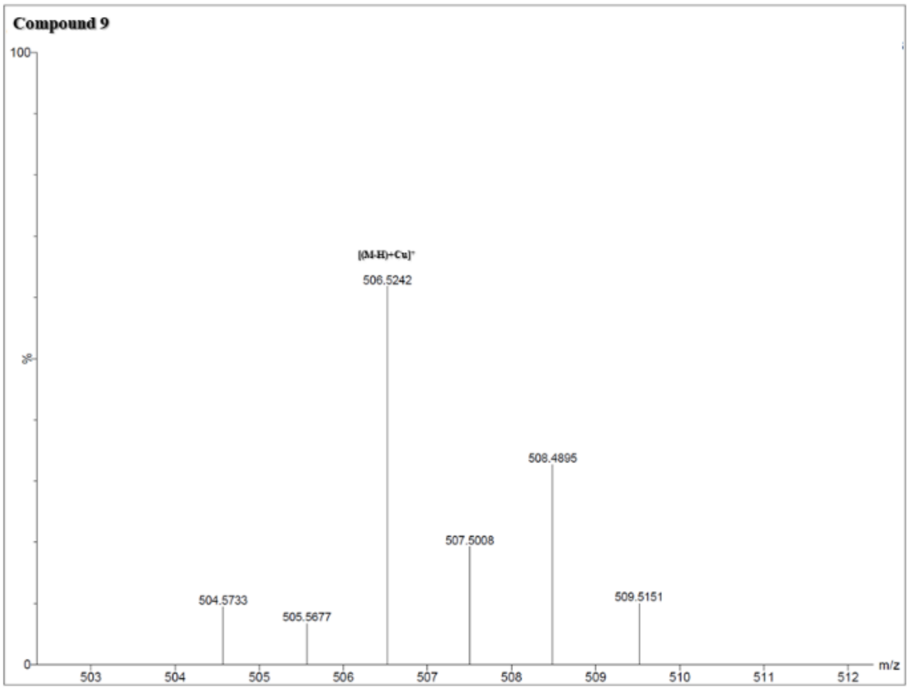

Figure S7. ESI-MS spectra of copper complexes of compounds **6-9.**
